# Biodiversity, seasonal abundance, and distribution of blackflies (Diptera: Simuliidae) in six different regions of Thailand

**DOI:** 10.1186/s13071-017-2492-y

**Published:** 2017-11-21

**Authors:** Wichai Srisuka, Hiroyuki Takaoka, Yasushi Otsuka, Masako Fukuda, Sorawat Thongsahuan, Kritsana Taai, Atiporn Saeung

**Affiliations:** 1 Entomology Section, Queen Sirikit Botanic Garden, P.O. Box 7, Chiang Mai, 50180 Thailand; 2 Institute of Biological Sciences, Faculty of Science, University of Malaya, 50603 Kuala Lumpur, Malaysia; 3 Research Center for the Pacific Islands, Kagoshima University, Kagoshima, 890-8580 Japan; 4 Division of Life Science Research, Research Promotion Institute, Oita University, Hasama, Oita 879-5593 Japan; 5 Faculty of Veterinary Science, Prince of Songkla University, Songkhla, 90110 Thailand; 6 Faculty of Veterinary Medicine, Western University, Kanchanaburi, 71170 Thailand; 7 Department of Parasitology, Faculty of Medicine, Chiang Mai University, Chiang Mai, 50200 Thailand

**Keywords:** Blackfly, *Simulium*, Biodiversity, Shannon diversity index, Regional distribution, Thailand

## Abstract

**Background:**

Blackflies are an important medical and veterinary group of small blood-sucking insects. Ninety-three blackfly species have been reported in Thailand. However, information on their biodiversity and population dynamics in each region is lacking. The main aim of this study was to assess the regional biodiversity, seasonal abundance and distribution of blackflies in six eco-geographically different regions in the country.

**Methods:**

Blackfly larvae and pupae were sampled monthly from 58 sites between May 2011 and April 2013. Diversity parameters, seasonal abundance, regional distribution and frequency of species occurrence in stream sites were analyzed.

**Results:**

A total of 19,456 mature larvae representing 57 species, and belonging to six subgenera in the genus *Simulium* Latreille (*s.l.*), were found. The five predominant taxa were *S*. *fenestratum* (8.6%), the *S. asakoae* complex (8.3%), *S. nakhonense* (7.5%), the *S. siamense* complex (7.4%) and the *S. doipuiense* complex (6.7%). The most frequent taxa at all sites were the *S. asakoae* complex (84.5%), followed by *S. fenestratum* (82.8%), the *S. siamense* complex (75.9%), *S. decuplum* (60.3%), *S. nakhonense* (58.6%) and the *S. tani* complex (48.3%). The richness of regional species was highest (40 species) in the north and predominated in the cold season. However, blackflies in the south predominated during the hot season. The highest numbers of blackflies collected from central, northeastern, eastern and western regions of the country were observed in the rainy season. Overall, the mean number of blackflies collected across the six regions during the rainy and cold season had no statistically significant difference, but it differed significantly in the hot season.

**Conclusions:**

Blackflies in Thailand were surveyed in all three seasons across six geographical regions. These findings demonstrated that blackfly communities at each stream site varied with seasonality, and the regional relative abundance of blackflies differed markedly in the hot season. It was also found that the occurrence and distribution of blackflies in each region were associated strongly with elevation.

**Electronic supplementary material:**

The online version of this article (10.1186/s13071-017-2492-y) contains supplementary material, which is available to authorized users.

## Background

Blackflies (Diptera: Simuliidae) are distributed widely in all zoogeographical regions and found almost everywhere with running water that is suitable as a habitat for their aquatic stages [1]. Larvae and pupae are aquatic, and attach themselves to various submerged objects in many types of lotic environments, ranging from large rivers to tiny spring-fed trickles, and from swift currents to water that barely moves [2]. The choice of habitat usually varies between species. Due to their blood-sucking habits, adult females of certain blackfly species are of a medical and veterinary importance. Blackflies have been considered as vectors of many pathogens, such as filarioid nematodes of the genus *Onchocerca* in humans, cattle and deer, the genus *Dirofilaria* in bears, the genus *Splendidofilaria* in ducks; blood protozoans of the genera *Leucocytozoon* and *Trypanosoma* in birds; and viruses (rift valley fever, vesicular stomatitis) in horses and cattle; as well as chlamydial bacteria that cause blindness in sheep and abortion in cattle [1–3]. Furthermore, blackfly bites can cause other severe problems in humans, since they frequently inflict pain, localized swelling, chronic dermatitis and inflammation accompanied by intense irritation that lasts for several days or even weeks [2].

In Thailand, a total of 93 blackfly species belonging to six subgenera, including *Asiosimulium*, *Daviesellum*, *Gomphostilbia*, *Montisimulium*, *Nevermannia* and *Simulium*, have been reported ([4], W. Srisuka, unpublished observations), with most new blackfly species being discovered in the northern part of the country. Remarkably, although the above information reflects rich species diversity, there are only a few reports of simuliids from other regions in Thailand, for instance, *S. otsukai*, *S*. *thongsahuani*, *S. datfaense* and *S. trangense*, in the south [5–7]; *S. vanellum* from the west [8], *S*. *atipornae* and *S. lomkaoense* from central Thailand [9, 10]; and *S. kuvangkadilokae* from the northeast [11, 12]. Notably, there were no reports on regional biodiversity, seasonal abundance or distribution of blackflies in macro-scale areas of Thailand, apart from only the hotspot area in the tropical rainforest at Doi Pha Hom Pok National Park, in the northern region [13]. Additionally, human-biting blackfly species are found in large numbers, and cause irritation in domestic environments and to indigenous people and tourists [14], thus, most previous studies focused on the annual biting activity of adult females at Doi Inthanon and Doi Suthep-Pui National Park as well as in the village of Ban Pang Faen, Chiang Mai Province, northern Thailand [14–17].

Hence, the main aim of this study was to determine the seasonal abundance and dynamics of blackflies in six geographically and ecologically different regions of Thailand.

## Methods

### Study areas and sampling

**Fig. 1:**
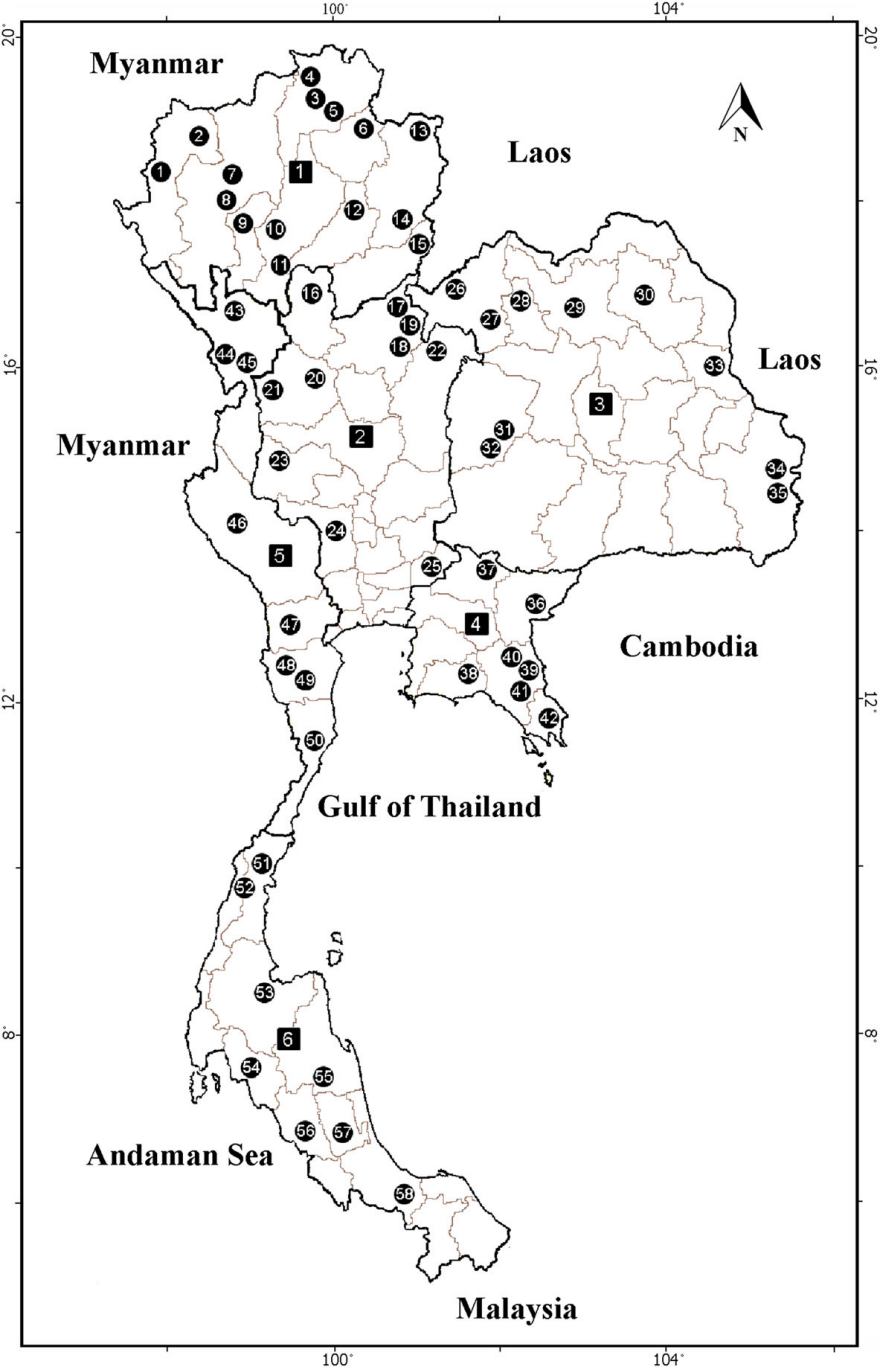
Map of Thailand showing 58 fixed-stream sites located in 41 provinces where larvae and pupae of blackflies were collected during the two-year study period (May 2011 to April 2013). Details of sampling sites are given in Additional file 1

This study was carried out at 58 fixed-stream sites in 41 provinces in six regions across Thailand, including 15, 10, 10, 7, 8 and 8 sites in the north (9 provinces), central (7 provinces), northeast (7 provinces), east (5 provinces), west (5 provinces) and south (8 provinces), respectively (Fig. 1, Additional file 1: Table S1). A total of 696 collections were made in this study (12 at each 58 fixed-stream sites at monthly intervals) from May 2011 to April 2013 which covered all seasons for each region. Larvae and pupae were hand sampled using fine forceps from available substrates in streams, such as fallen leaves, mud or rock surfaces, and trailing grasses. Forty-five minutes exactly were spent for the collection of larvae and pupae by the same person (one person) at each stream site. Larvae were preserved in 80% ethanol. The substrates were cut into pieces so that each part harboured a single pupa. Matured pupae were maintained individually in a plastic tube (10 cm long and 1.7 cm in diameter) with very little water at the bottom until adults emerged. After emergence, adult flies were kept alive in the same tube for at least 24 h, to secure hardening and colouring of their body and legs. Adult flies, associated with their pupal exuviae, were used to confirm the species identification of the larvae.

### Meteorology and regions

The classification of the season in each region follows the Thai Meteorological Department, which bases its records on rainfall and air temperature data. Thus, each year is divided climatically into three seasons in the following regions: North region, with hilly and mountainous areas ranging from 392 to 2210 m in height, total rainfall of approximate 1287 mm and an average air temperature of 25 °C; West region, with mostly mountainous areas in the range of 111–560 m in height, similar to the north, and total rainfall and average air temperature of 1243 mm and 27.13 °C, respectively; Central region, with largely low-level plains and a few mountains ranging from 167 to 1550 m high in the northern and western part of the region, with a total rainfall and average air temperature of 1377 mm and 26.1 °C, respectively; Northeast region, which is a naturally high-level plain called the northeast plateau; Northwest-southeast region, which has Phu Phan Ridge oriented in the northeastern portion that separates this area into two basins, the first one is a large high-level plain in the west and the other smaller and sloped towards the east with an elevation ranging from 110 to 1337 m; this region has a total rainfall and average air temperature of 1589 mm and 25.1 °C, respectively; East region, which is mountainous in the northeast, and its eastern area is close to the Gulf of Thailand with an elevation ranging from 76 to 409 m, and total rainfall and an average air temperature of 2903 mm and 26.7 °C, respectively; South region, which has a peninsula mountain spine that is very steep. Its eastern area is close to the Gulf of Thailand and its western region near the Andaman Sea. It has an elevation ranging from 111 to 560 m, and total rainfall and an average air temperature of 2601 mm and 28.9 °C, respectively.

### Species identification

Species identification was based on morphological characteristics of last-instar larvae (matured), pupae and reared adults by using the standard keys of Takaoka & Choochote [18], which covered 45 blackfly species, and additional keys that dealt with blackflies in Thailand [5–12, 19–26]. When formally named species were known to consist of cryptic species, they were referred to as species complex [4]. All specimens of blackflies from this study were deposited at the Entomology Section, Queen Sirikit Botanic Garden (QSBGE), Chiang Mai Province, Thailand.

### Data analysis

Species and relative abundance of mature larvae at each site were recorded. The frequency of blackfly species was calculated by the total number of species occurrence divided by the total number of collections and presented in percentages. Stream occurrence (SO) (expressed in percentage) was obtained by calculating the number of sites where a species was taken and dividing it by the total number of sites sampled (*n* = 58). Species diversity and richness (SDR) version 4 [27] and PAST version 3.11 were employed for statistical analyses [28]. Determination and comparison of diversity parameters between regions were calculated using the Shannon-Wiener index (H), the expected value of H (Exp H) and evenness (J’). The species accumulation curves (rarefaction) for regions were also compared. In evaluating species richness, the first order jackknife was used to estimate the number of species presenting in all stream sites. Sample interpopulation was used to estimate the number of species from all regions (696 collections) [27]. Detrended correspondence analysis (DCA) was used to describe the regional distribution of blackfly larvae associated with sampling sites [28]. A ternary plot, based on data of blackfly species in each season and region, was used to interpret seasonal occurrence and abundance [28]. Regional and seasonal differences in blackflies were compared using non-parametric Kruskal-Wallis tests, and *P*-values were adjusted by the Bonferroni correction for *post-hoc* multiple comparison tests. In addition, the Mann-Whitney *U*-test was used to compare the mean number of blackflies collected from the southern region in two seasons. Statistical analyses were conducted using IBM SPSS statistics, version 24 for Windows (Chicago, SPSS Inc.). Statistical significance was set at *P* < 0.05.

## Results

### Species composition of blackflies

A total of 19,456 mature larvae, representing 57 blackfly species of six subgenera, were collected from 58 stream sites across six regions in Thailand (Table 1, Additional file 1: Table S1). At the subgenus level, *Simulium* was the most diverse (28 species), followed by *Gomphostilbia* (16 species), *Nevermannia* (7 species), *Asiosimulium* (3 species), *Montisimulium* (2 species) and *Daviesellum* (1 species). *Simulium fenestratum*, the *S. asakoae* complex, *S. nakhonense*, the *S. siamense* complex, and the *S. doipuiense* complex were the five predominant taxa, each representing 8.6% (*n* = 1681), 8.3% (*n* = 1608), 7.5% (*n* = 1451), 7.4% (*n* = 1441) and 6.7% (*n* = 1298) of those collected. The most frequent taxa at all sites were the *S. asakoae* complex (84.5%, 49/58 sites), followed by *S. fenestratum* (82.8%, 48/58 sites), the *S. siamense* complex, (75.9%, 44/58 sites), *S. decuplum* (60.3%, 35/58 sites), *S. nakhonense* (58.6%, 34/58 sites) and the *S*. *tani* complex, (48.3%, 28/58 sites).

**Table 1:** Total number, relative abundance (percentage), and stream occurrence (SO) of mature larvae of 57 blackfly species collected from 58 sampling sites in six regions in Thailand

Species	Total collected	%flies	%SO
*Simulium* (*Asiosimulium*) *furvum*	34	0.2	1.7
*Simulium* (*Asiosimulium*) *oblongum*	487	2.5	17.2
*Simulium* (*Asiosimulium*) *wanchaii*	34	0.2	3.5
*Simulium* (*Daviesellum*) *pahangense*	9	0.1	5.2
*Simulium* (*Gomphostilbia*) *angulistylum* complex	515	2.7	22.4
*Simulium* (*Gomphostilbia*) *asakoae* complex^a,b^	1608	8.3	84.5
*Simulium* (*Gomphostilbia*) *burtoni*	523	2.7	24.1
*Simulium* (*Gomphostilbia*) *chiangdaoense*	515	2.7	10.3
*Simulium* (*Gomphostilbia*) *chumpornense*	182	0.9	27.6
*Simulium* (*Gomphostilbia*) *curtatum*	235	1.2	12.1
*Simulium* (*Gomphostilbia*) *decuplum* ^a^	1175	6	60.3
*Simulium* (*Gomphostilbia*) *dentistylum*	367	1.9	37.9
*Simulium* (*Gomphostilbia*) *duolongum*	450	2.3	22.4
*Simulium* (*Gomphostilbia*) *gombakense*	90	0.5	13.8
*Simulium* (*Gomphostilbia*) *inthanonense*	753	3.9	19
*Simulium* (*Gomphostilbia*) *piroonae*	52	0.3	1.7
*Simulium* (*Gomphostilbia*) *kuvangkadilokae*	98	0.5	3.5
*Simulium* (*Gomphostilbia*) *parahiyangum*	3	0	1.7
*Simulium* (*Gomphostilbia*) *sheilae*	556	2.9	44.8
*Simulium* (*Gomphostilbia*) *siamense* complex^a,b^	1441	7.4	75.9
*Simulium* (*Montisimulium*) *nanense*	51	0.3	1.7
*Simulium* (*Montisimulium*) sp.	57	0.3	1.7
*Simulium* (*Nevermannia*) *aureohirtum*	408	2.1	22.4
*Simulium* (*Nevermannia*) *fangense*	22	0.1	1.7
*Simulium* (*Nevermannia*) *feuerborni* complex	142	0.7	8.6
*Simulium* (*Nevermannia*) *fruticosum*	329	1.7	17.2
*Simulium* (*Nevermannia*) *khunklangense*	73	0.4	1.7
*Simulium* (*Nevermannia*) *maeaiense*	241	1.2	8.6
*Simulium* (*Nevermannia*) *vessabutrae*	7	0	1.7
*Simulium* (*Simulium*) *atipornae*	98	0.5	3.5
*Simulium* (*Simulium*) *baimaii*	69	0.4	1.7
*Simulium* (*Simulium*) *brevipar*	16	0.1	1.7
*Simulium* (*Simulium*) *bullatum*	61	0.3	10.3
*Simulium* (*Simulium*) *chamlongi*	217	1.1	20.7
*Simulium* (*Simulium*) *chiangmaiense*	79	0.4	3.5
*Simulium (Simulium) doipuiense* complex^b^	1298	6.7	25.9
*Simulium* (*Simulium*) *fenestratum* ^a,b^	1681	8.6	82.8
*Simulium* (*Simulium*) *grossifilum*	15	0.1	3.5
*Simulium* (*Simulium*) *lampangense*	139	0.7	5.2
*Simulium* (*Simulium*) *lomkaoense*	98	0.5	3.5
*Simulium* (*Simulium*) *malayense*	21	0.1	1.7
*Simulium* (*Simulium*) *manooni*	196	1	8.6
*Simulium* (*Simulium*) *nakhonense* ^a,b^	1451	7.5	58.6
*Simulium* (*Simulium*) *nigrogilvum*	17	0.1	5.2
*Simulium* (*Simulium*) *nobile*	843	4.3	17.2
*Simulium* (*Simulium*) *nodosum*	301	1.5	15.5
*Simulium* (*Simulium*) *phayaoense*	54	0.3	6.9
*Simulium* (*Simulium*) *prayongi*	35	0.2	1.7
*Simulium* (*Simulium*) *phukaense*	37	0.2	6.9
*Simulium* (*Simulium*) *quinquestriatum*	614	3.2	34.1
*Simulium* (*Simulium*) *siripoomense*	19	0.1	1.7
*Simulium* (*Simulium*) *takense*	69	0.4	1.7
*Simulium* (*Simulium*) *tani* complex^a^	385	2	48.3
*Simulium* (*Simulium*) *thailandicum*	228	1.2	12.1
*Simulium* (*Simulium*) *weji*	609	3.1	6.9
*Simulium* (*Simulium*) *yongi*	38	0.2	3.5
*Simulium* (*Simulium*) *yuphae*	311	1.6	36.2
Total	19,456	100	

^a^The most frequent taxa at all sites

^b^The most predominant taxa

### Species diversity, richness and distribution pattern

**Fig. 2:**
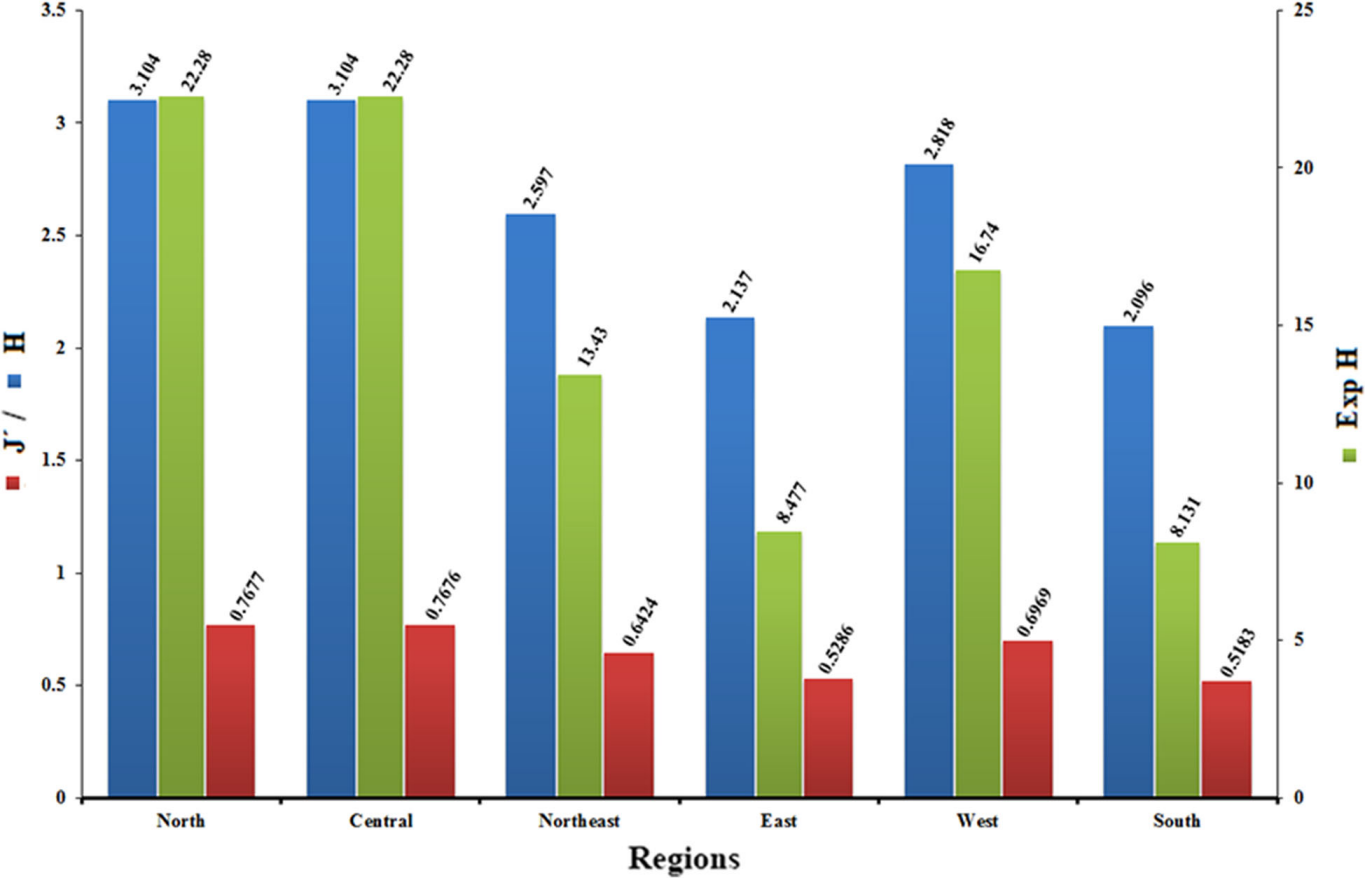
Diversity parameters, Shannon-Wiener index (H), evenness (Pielou J’) and expected H value (Exp H) for blackflies in the six regions of Thailand

Species diversity and richness of blackflies in each region are shown in Fig. 2, with the highest in the northern region (*H* = 3.1, *J’* = 0.8) and lowest in the southern (*H* = 2.1, *J’* = 0.5). Of 58 stream sites, the Shannon diversity index (H) was highest at Rom Klao (Phitsanulok Province), followed by Doi Phu Kha (Nan Province), Phu Ruea (Loei Province) and Mae Wong (Kamphaeng Phet Province), which represented 2.4, 2.4, 2.3 and 2.3, respectively (Additional file 2: Table S2).

**Fig. 3:**
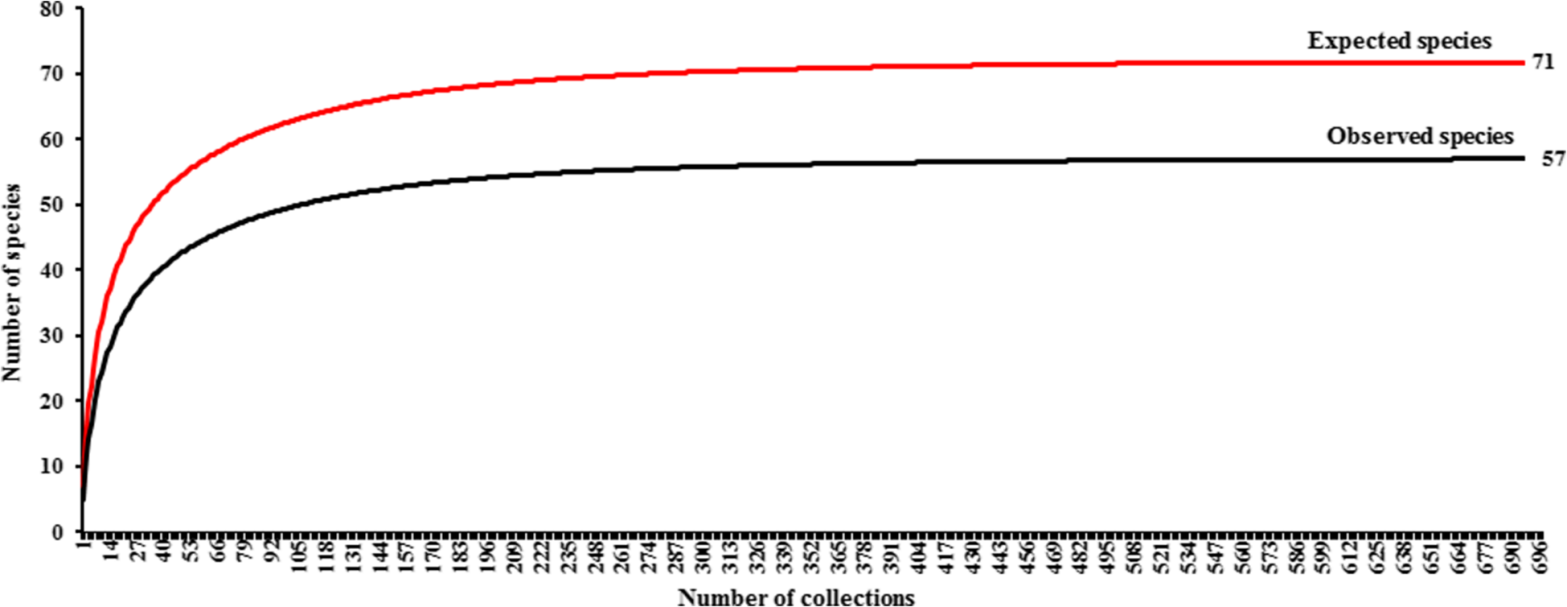
Species accumulation (rarefaction) curves for blackflies from 696 collections overall at 58 sites in the six regions of Thailand

**Fig. 4:**
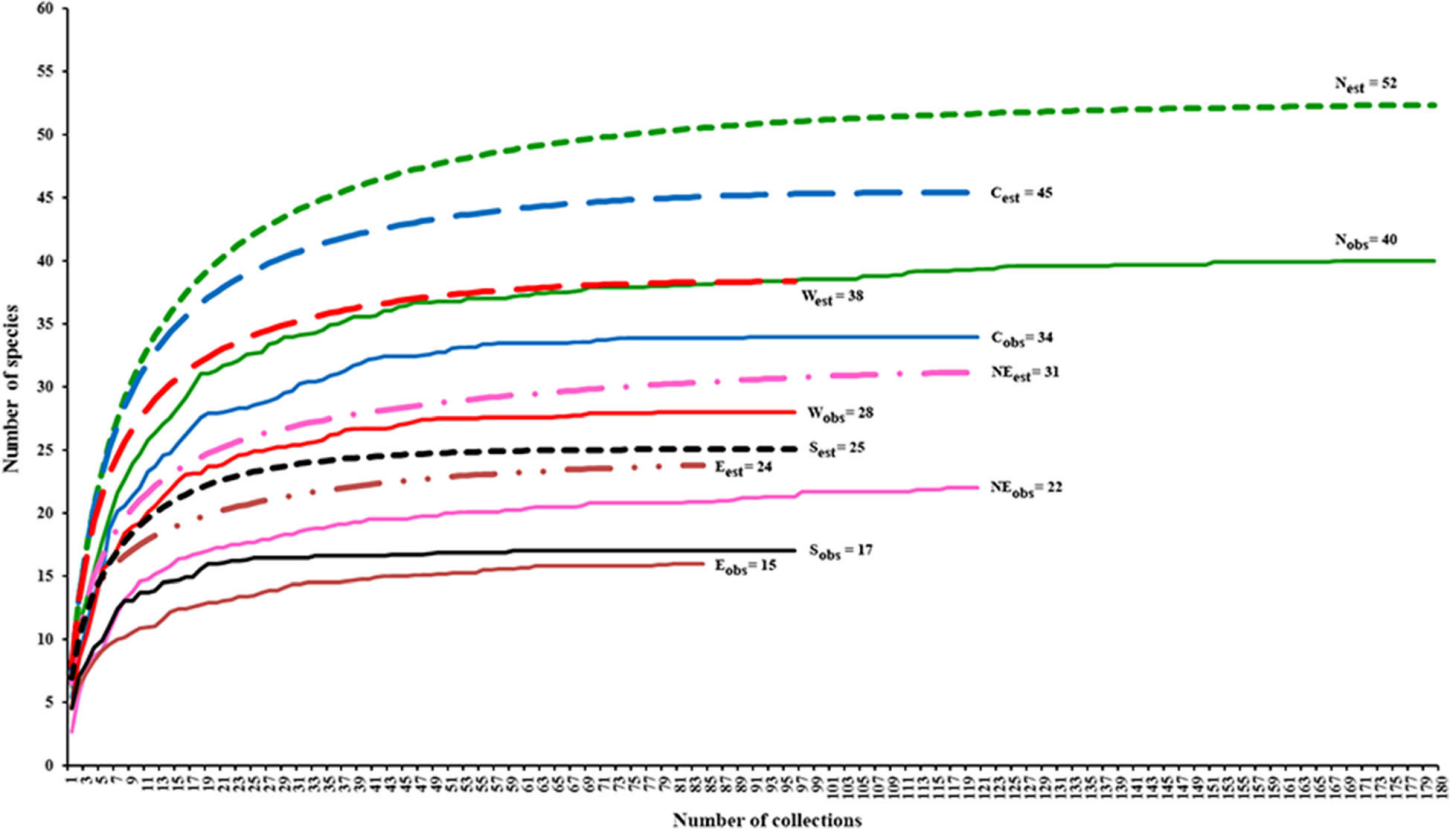
Species accumulation and species richness curves representing the observed (obs) and estimated (est) number of blackflies collected from 58 sites across the six regions of Thailand (northern: 180 collections; central: 120 collections; northeastern: 120 collections; eastern: 84 collections; western: 96 collections; southern: 96 collections)

The species accumulation (rarefaction) curves (Fig. 3) showed the expected and observed richness of species occurring in all sites, with a total from all collections being 71 and 57 species, respectively. The expected (± SE) and observed species richness were 52 ± 4.5 and 40 spp. in northern, 45 ± 4.1 and 34 spp. in the central, 38 ± 2.9 and 28 spp. in western, 31 ± 2.8 and 22 spp. in northeastern, 25 ± 4.3 and 17 spp. in southern and 24 ± 1.8 and 15 spp. in eastern regions, respectively (Fig. 4).

**Fig. 5:**
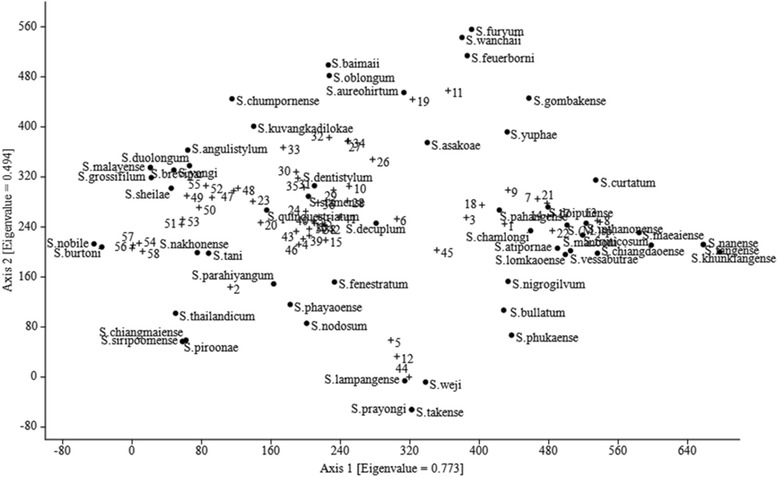
Ordination diagrams extracted by detrended correspondence analysis (DCA) of blackflies distributed in 58 sites

The detrended correspondence analysis (DCA) for the distribution of blackfly species associated with sampling sites (axis 1: eigenvalue 0.8; axis 2: eigenvalue 0.5) is presented in Fig. 5. Overall, the *S. asakoae* complex and *S. fenestratum* were common species found in all sites. The distribution of the *S. doipuiense* complex, *S. inthanonense*, *S. fruticosum*, *S. chiangdaoense*, *S. maeaiense*, *S.* (*Montisimulium*) sp., *S. vessabutrae*, *S. atipornae* and *S. lomkaoense* were associated strongly with high elevation (site nos. 8, 13,14, 17, 21 and 22) in the northern and central region, while *S. quinquestriatum*, *S. siamense* complex, *S. decuplum* and *S. dentistylum* were associated with low elevation (site nos. 20, 23, 24, 25, 29, 30, 31, 35, 38, 40, 41 and 42) in the central and northeastern regions. However, *S. lampangense*, *S. weji, S. prayongi* and *S. takense* were associated with calcareous waterfalls (site nos. 5, 12 and 44).

### Seasonal dynamics

**Fig. 6:**
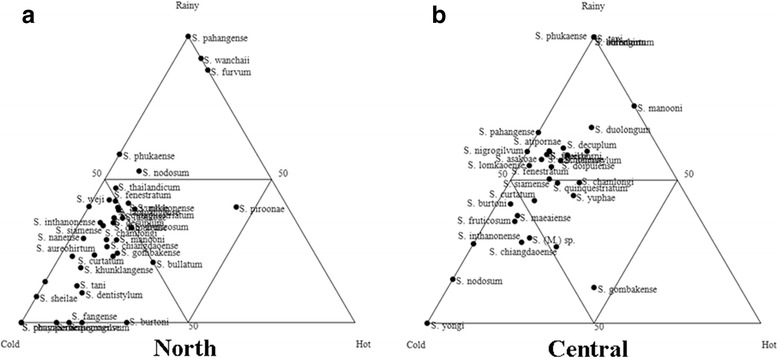
Ternary plots representing the occurrence and seasonal abundance of blackflies in the northern (**a**) and central region (**b**) of Thailand

**Fig. 7:**
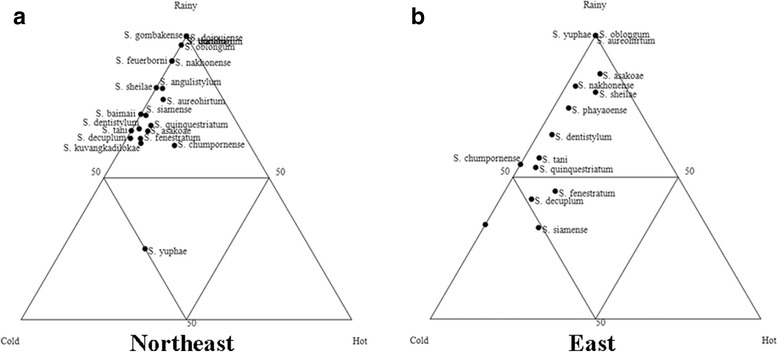
Ternary plots representing the occurrence and seasonal abundance of blackflies in the northeastern (**a**) and eastern region (**b**) of Thailand

**Fig. 8:**
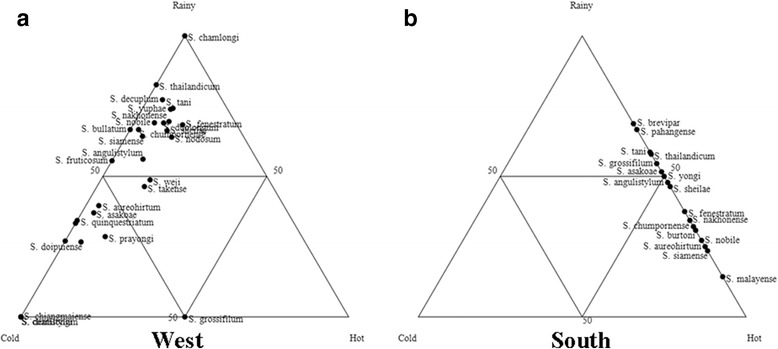
Ternary plots representing the occurrence and seasonal abundance of blackflies in the western (**a**) and southern region (**b**) of Thailand

Overall, *S*. *fenestratum* was the dominant species during all three seasons (Additional file 3: Table S3). Almost all of the species in the north were dominant in the cold season, and *S. chumpornense* and *S. phayaoense* were recorded only during that time, while *S. pahangense* was collected only during the rainy season (Fig. 6, Additional file 3: Table S3). Most species in the central region were discovered in the rainy season, and *S. oblongum*, *S. aureohirtum*, *S. bullatum*, *S. phukaense* and the *S. tani* complex were found only during that time, whereas *S. yongi* was only recorded in the cold season (Fig. 6, Additional file 3: Table S3). A greater number of blackflies were collected during the rainy season in the northeastern, eastern and western regions (Figs. 7, 8, Additional file 3: Table S3). *Simulium oblongum*, *S. aureohirtum* and *S*. *yuphae* were found only during the rainy season in the eastern region, similar to species found in the central region. Remarkably, blackflies in the southern region were more dominant during the hot season (Fig. 8, Additional file 3: Table S3). The mean number of blackflies collected across the six regions during the rainy (Kruskal-Wallis test, *H* = 6.242, *df* = 5, *P* = 0.283) and cold season (Kruskal-Wallis test, *H* = 8.650, *df* = 4, *P* = 0.070) had no statistically significant difference, but it differed significantly in the hot season (Kruskal-Wallis test, *H* = 26.589, *df* = 5, *P* < 0.0001).

### Regional relative abundance

#### Northern region

The most frequent taxa at all sites were *S. asakoae* complex (80%) and *S. fenestratum* (80%), followed by *S*. *yuphae* (73.3%) and the *S. siamense* complex, (66.7%). In addition, the *S. doipuiense* complex, *S*. *inthanonense*, the *S. asakoae* complex, *S. decuplum/S. fenestratum* and *S. chiangdaoense* were the five predominant taxa, representing 15.4% (*n* = 860), 11.6% (*n* = 647), 7.0% (*n* = 389), 6.4% (*n* = 358) and 5.3% (*n* = 298), respectively (Additional file 4: Table S4). The hot season had significantly lower mean numbers when compared to the rainy (Kruskal-Wallis test, *H* = 21.195, *df* = 2, *P* = 0.021) and cold seasons (Kruskal-Wallis test, *H* = 34.122, *df* = 2, *P* < 0.0001).

#### Central region

The most frequent taxa at all sites were *S. asakoae* complex (90%) and the *S. siamense* complex, (90%), followed by *S. decuplum* and *S. fenestratum* (80%). Additionally, the *S. asakoae* complex, the *S*. *doipuiense* complex, the *S*. *siamense* complex, *S. nakhonense* and *S. ducuplum*, were the five predominant taxa, representing 10.3% (*n* = 353), 10% (*n* = 345), 7.4% (*n* = 254), 7.3% (*n* = 251) and 6.3% (*n* = 218), respectively (Additional file 5: Table S5). There were significant differences in the mean number of blackflies captured in the central region during the hot season, when compared to the rainy (Kruskal-Wallis test, *H* = 30.265, *df* = 2, *P* < 0.0001) and cold seasons (Kruskal-Wallis test, *H* = 19.456, *df* = 2, *P* = 0.020).

#### Northeastern region

The most frequent taxa at all sites were *S. asakoae* complex and *S. siamense* complex (100%), followed by *S. decuplum*, *S. fenestratum* and *S. quinquestriatum* (90%). In addition, the *S. asakoae* complex, *S. oblongum*, *S. fenestratum*, the *S. siamense* complex and *S. quinquestriatum* were the five predominant taxa, representing 15.7% (*n* = 415), 13.4% (*n* = 353), 12.8% (*n* = 338), 11% (*n* = 290) and 8.3% (*n* = 219), respectively (Additional file 6: Table S6). There was a significant difference in the mean number of blackflies collected among the three seasons (Kruskal-Wallis test, *H* = 28.687, *df* = 2, *P* < 0.0001).

#### Eastern region

The most frequent taxa at all sites were *S. decuplum*, *S. dentistylum*, the *S. siamense* complex, *S. fenestratum* and the *S. tani* complex (100%), followed by the *S*. *asakoae* complex and *S. nakhonense* (85.7%). Additionally, *S. fenestratum*, the *S*. *siamense* complex, *S. decuplum*, *S. dentistylum* and *S. nakhonense* were the five predominant taxa, representing 24% (*n* = 479), 19.6% (*n* = 391), 18.5% (*n* = 369), 8.1% (*n* = 163) and 7% (*n* = 140), respectively (Additional file 7: Table S7). The mean number of blackflies captured in the hot season was lower than that in the rainy (Kruskal-Wallis test, *H* = 21.195, *df* = 2, *P* = 0.021) and cold season (Kruskal-Wallis test, *H* = 34.122, *df* = 2, *P* < 0.0001). There was a significant difference in a mean number of the blackflies collected in this region between the hot and rainy seasons (Kruskal-Wallis test, *H* = 15.033, *df* = 2, *P* < 0.005).

#### Western region

The most frequent taxa at all sites were *S. nakhonense* and *S. tani* complex (75%), followed by the *S. angulistylum* complex, the *S. asakoae* complex, *S. sheilae*, the *S. siamense* complex and *S. fenestratum* (62.5%). In addition, *S. nakhonense*, *S. weji*, the *S. angulistylum* complex, *S. duolongum* and the *S. siamense* complex were the five predominant taxa, representing 16.1% (*n* = 548), 12% (*n* = 406), 9.3% (*n* = 317), 7.5% (*n* = 255) and 6.5% (*n* = 221), respectively (Additional file 8: Table S8). The mean number of blackflies captured was lower during the hot season than that during the rainy (Kruskal-Wallis test, *H* = 27.071, *df* = 2, *P* < 0.0001) and cold season (Kruskal-Wallis test, *H* = 22.589, *df* = 2, *P* = 0.002).

#### Southern region

The most frequent taxa at all sites were *S. burtoni*, *S. sheilae*, *S. nakhonense* and *S. nobile* (100%), followed by the *S. asakoae* complex and *S. fenestratum* (87.5%). Additionally, *S. nobile*, *S. burtoni*, *S. nakhonense*, *S. sheilae* and the *S. asakoae* complex were the five predominant taxa, representing 31.1% (*n* = 746), 19.8% (*n* = 474), 12.4% (*n* = 297), 12.1% (*n* = 289) and 5.2% (*n* = 124), respectively (Additional file 9: Table S9). There were no significant differences in the mean number of blackflies captured between the hot and rainy seasons (Mann-Whitney *U*-test, *U* = 128.000, *P* = 0.570).

## Discussion

### Species composition, species richness, seasonal abundance and diversity

The number of blackflies reached its highest during the cold season, according to a previous report by Srisuka et al. [13], who studied the seasonal biodiversity of blackflies at Doi Pha Hom Pok, northern Thailand. The greatest number of blackflies in the southern region was in the hot season. This study found that seven of seventeen species identified from this region increased their populations approximately two to three times during this season. This observation agrees with the study of blackflies in northern Sweden, where they were higher in the summer than other seasons [29]. The highest number of blackflies collected from central, northeastern, eastern and western regions of Thailand peaked in the rainy season. The findings in this study were consistent with those in a previous report by Pramual & Wongpakam [30], who studied the seasonal variation of blackflies at Phu Phan mountain range in northeastern Thailand. They demonstrated that the species abundance was higher in rainy seasons than in others and blackfly communities at each stream site varied with seasonality, i.e. *S. nakhonense*, the *S. angulistylum* complex and *S. kuvangkadilokae* were more dominant in the rainy season, whereas the *S*. *asakoae* complex, *S. aureohirtum* and *S. trangense* were dominant in the hot and cold season [30]. Likewise, blackflies were caught in higher numbers during the rainy season in Nigeria, Africa [31]. Takaoka [32] showed that seasonal abundance patterns of adult populations of *S. ochraceum*, the vector of onchocerciasis in Guatemala, Central America, differ by localities depending on the availability of permanent and temporary streams suitable for its immature stages.

In addition to seasons and geographical locations, elevation also can influence blackfly populations. The results of this study showed that the Shannon diversity index was highest in areas with high elevations, i.e. Rom Klao (1047 m), Doi Phu Kha (1629 m), Phu Ruea (1337 m) and Mae Wong (1274 m). It was found that 36 species manifested in optimal or unique environments that had suitable factors for their breeding habitats. For example, *S. baimaii* breeds at Phu Kradueng, Loei Province in only high mountains, with slow-flowing streams exposed to sunlight. Likewise, all species members in the subgenus *Montisimulium* are restricted to high elevations at Doi Inthanon National Park, Chiang Mai Province. In contrast, *S. gombakense* has a wide vertical distribution range from a height of 500 m in small streams in the foothills to 2100 m near the summit of Doi Pha Hom Pok National Park [13], and it is also found at an elevation of 412 m at Mae Klang Waterfall, Doi Inthanon National Park [33]. In addition, the *S*. *asakoae* complex, *S*. *fenestratum*, the *S*. *siamense* complex, *S. decuplum*, *S*. *nakhonense*, and the *S*. *tani* complex were the most common taxa found in this study, which is similar to previous reports by Pramual & Kuvangkadilok [34], and Pramual & Wongpakam [30].

### Relationship of subgenera to elevation

The subgenus *Asiosimulium* is a small and endemic subgenus in the Oriental region. It is represented by four species, of which three, *S. oblongum*, *S. wanchaii* and *S. furvum*, have been reported in Thailand [35–37], and the remaining one, *S. suchitrae*, in Nepal [38]. The first three species were found in lowland streams, flowing slowly over rock surfaces exposed to the sun during the rainy season, while *S. suchitrae* was found at high elevation (1826 m) in a small stream flowing slowly over rocks [38]. Both *S. furvum* and *S. wanchaii* were restricted to their sites, but *S. oblongum* was distributed widely in and near the northeastern, central and eastern regions. The subgenus *Daviesellum* is represented by two species, *S. pahangense* and *S. courtneyi*, in Thailand [39]. Only *S. pahangense* was distributed at high elevation from northern to central regions along the boundary with Myanmar, and also in lowland streams in the southern region. Most species of *Gomphostilbia*, such as those of the *S. batoense*, *S. ceylonicum*, *S. epistum*, *S. gombakense* and *S. varicorne* species-groups, are the second largest subgenus in Thailand and distributed at low elevations. This study found *S. sheilae*, the *S. siamense* complex and *S. chumpornense* in all six regions of Thailand, with their breeding habitats mostly in lowland streams as previously reported [7, 30]. In contrast, species of the *S. asakoae* and *S. darjeelingense* species-groups were found in highland streams, except for the *S. asakoae* complex, which was distributed widely from low elevations to 2500 m at the summit of Doi Inthanon National Park, Chiang Mai Province and in other Asian countries, such as Malaysia, China (Hong Kong) and Vietnam [7, 13, 21, 25, 40–42]*.*

The subgenus *Montisimulium* is represented by six species in Thailand. Two of them, i.e. *S. nanense* and *S*. (*Montisimulium*) sp., were discovered in this study at high elevations, as reported by Takaoka & Somboon [43] and Takaoka et al. [44], who collected three species of this subgenus at high elevations ranging from 2229 to 3720 m in Bhutan and 1750 m in Vietnam, respectively. The remaining species have been found only on Doi Inthanon and Doi Pha Hom Pok in Chiang Mai Province [45, 46]. Of ten species of the subgenus, *Nevermannia* reported in Thailand, seven were found in this study. Most species were collected at high elevations, for example, the high mountains of Chiang Mai Province, northern Thailand [13, 26, 47, 48]. Other reports from several other Asian countries, such as Malaysia, Myanmar, Vietnam, Indonesia and Bhutan, indicated that members of this subgenus were associated with high elevations ranging from 1000 to 2532 m [43, 49–53]. The subgenus *Simulium* is the largest subgenus in Thailand, including 45 described species, of which 28 (62% of total species) were collected. Most of the common taxa, such as *S. fenestratum*, *S. nakhonense*, *S. quinquestriatum*, and the *S. tani* complex, breed in lowland streams. The findings of this study were in accordance with those reported by Takaoka et al. [44], Srisuka et al. [13] and Pramual & Wongpakham [30]. Species of the *S. christophersi*, *S. malyschevi* and *S. variegatum* species-groups were distributed in middle to high elevations (1200–2200 m), as previously studied in Vietnam and Thailand [44, 54]. In contrast, most of the species within the *S. multistriatum*, *S. nobile* and *S. striatum* species-groups occurred in lowland streams, as reported by Srisuka et al. [13] and Ishii et al. [17]. Members of the *S. griseifrons* species-group colonized streams at low to high elevations (200–2210 m).

## Conclusion

The findings of this study demonstrated that the richness and relative abundance of blackflies were different between regions, and blackfly communities at each stream site varied with seasonality. Also, the elevation of sampling sites, which ranged from high mountainous to lowland streams as well as covering all mainland steams, influenced the distribution of blackflies in the country. Concurrent species, population dynamics and seasonal abundance in each area are important as useful information for pest species management and control programs, and especially for ecotourism in forests, by waterfalls and in high mountainous areas, where the number of tourists increases yearly.

## Additional files

Additional file 1: Table S1. Names of sampling sites, geographical coordinates, altitudes, and environmental variables for blackfly collections at 58 sampling sites in six regions of Thailand. (DOCX 38 kb)

Additional file 2: Table S2. Diversity parameters for blackflies at 58 sampling sites in the six regions of Thailand. (DOCX 19 kb)

Additional file 3: Table S3. Seasonal abundance and species richness of blackfly species at 58 sampling sites representing six regions in Thailand. (DOCX 44 kb)

Additional file 4: Table S4. Regional distribution and relative abundance of blackflies at 15 sampling sites in northern Thailand. (DOCX 30 kb)

Additional file 5: Table S5. Regional distribution and relative abundance of blackflies at 10 sampling sites in central Thailand. (DOCX 24 kb)

Additional file 6: Table S6. Regional distribution and relative abundance of blackflies at 10 sampling sites in northeastern Thailand. (DOCX 20 kb)

Additional file 7: Table S7. Regional distribution and relative abundance of blackflies at 7 sampling sites in eastern Thailand. (DOCX 18 kb)

Additional file 8: Table S8. Regional distribution and relative abundance of blackflies at 8 sampling sites in western Thailand. (DOCX 21 kb)

Additional file 9: Table S9. Regional distribution and relative abundance of blackflies at 8 sampling sites in southern Thailand. (DOCX 19 kb)

## Abbreviations

DCA: Detrended correspondence analysis
Exp H: Expected value of H; Pielou J’: evenness
H: Shannon-Wiener index
