# Supplementary material for: Biodiversity, seasonal abundance, and distribution of blackflies (Diptera: Simuliidae) in six different regions of Thailand
Source: Parasit Vectors. 2017 Nov 21;10:574. doi: 10.1186/s13071-017-2492-y (PMC5697434; doi:10.1186/s13071-017-2492-y)
Supplement: Supplementary file 1 — Names of sampling sites, geographical coordinates, altitudes, and environmental variables for blackfly collections at 58 sampling sites in six regions of Thailand. (DOCX 38 kb) [file 13071_2017_2492_MOESM1_ESM.docx]

**Additional file 1: Table S1.** Names of sampling sites, geographical coordinates, altitudes, and environmental variables for blackfly collections at 58 sampling sites in six regions of Thailand

| **Province** | **Code** | **Names of sampling sites** | **Regions*** | **Latitude/Longitude** | **Elevation**  **(m)** | **Streambed**  **particle size** | **Canopy cover** |
| --- | --- | --- | --- | --- | --- | --- | --- |
| Mae Hong Son | 1 | Namtok Mae Surin | N | 18°57'44.4"N 98°04'48.40"E | 1,094 | sand | partial |
|  | 2 | Huai Mae Sa | N | 19°28'34.1"N 98°17'34.90"E | 452 | sand | open |
| Chiang Rai | 3 | Doi Chang | N | 19°49'12.8"N 99°30'28.9"E | 621 | sand/small stone | open |
|  | 4 | Huai Kang Pla Waterfall | N | 20°05'15.7"N 99°46'49.1"E | 551 | sand | partial |
|  | 5 | Phu Kaeng Waterfall | N | 19°26'29.8"N 99°41'56.4"E | 532 | calcareous | partial |
| Phayao | 6 | Phu Sang | N | 19°42'43.2"N 100°23'40.6"E | 883 | silt | partial |
| Chiang Mai | 7 | Doi Suthep | N | 18°49'04.4"N 98°53'05.70"E | 1,356 | sand | complete |
|  | 8 | Ban Khun Klang | N | 18°33'29.4"N 98°28'51.7"E | 2,210 | sand | partial |
| Lamphun | 9 | Doi Khun Tan | N | 18°29'34.8"N 99°18' 02.8"E | 1,343 | silt/sand | partial |
| Lampang | 10 | Khun Tan service area | N | 18°23'27.2"N 99°13'00.1"E | 441 | sand | open |
|  | 11 | Mae Wa | N | 17°37'19.7"N 99°18'49.0"E | 468 | sand | partial |
| Phrae | 12 | Huai Rong Waterfall | N | 18°26'32.3"N 100°27'01.1"E | 392 | calcareous | partial |
| Nan | 13 | Doi Phu Kha | N | 19°10'49.9"N 101°06'26.4"E | 1,629 | silt | partial |
|  | 14 | Khun Sathan | N | 18°16'46.4"N 100°30'15.0"E | 1,288 | silt | partial |
| Uttaradit | 15 | Phu Soi Dao Waterfall | N | 17°42'19.2"N 100°57'07.8"E | 659 | stone | open |

| **Province** | **Code** | **Names of sampling sites** | **Regions*** | **Latitude/Longitude** | **Elevation**  **(m)** | **Streambed**  **particle size** | **Canopy cover** |
| --- | --- | --- | --- | --- | --- | --- | --- |
| Sukhothai | 16 | Tad Dean Waterfall | C | 17°33'19.0"N 99°29'35.1"E | 176 | stone | open |
| Phitsanulok | 17 | Rom Klao | C | 17°36’39.2"N 100°54’14.4"E | 1,047 | silt | partial |
|  | 18 | Maeka Muennoi Waterfall | C | 16°59’22.6"N 100°59’53.0"E | 1,310 | rock | open |
|  | 19 | Lan Hin Pum | C | 16°59’52.7"N 101°00’34.5"E | 1,228 | rock | open |
| Kamphaeng Phet | 20 | Klong Maepert | C | 16°02’40.3"N 99°17’27.3"E | 167 | stone/sand | open |
|  | 21 | Mae Wong | C | 16°05’21.2"N 99°06’47.9"E | 1,274 | silt/sand | complete |
| Phetchabun | 22 | Man Dang | C | 16°56'27.0"N 101°02'25.4"E | 1,550 | rock | complete |
| Uthai Thani | 23 | Huai Rabum | C | 15°29'36.3"N 99°23'55.2"E | 175 | sand | open |
| Suphan Buri | 24 | Pu Toei | C | 14^o^56'28.9''N 99^o^26'56.8''E | 551 | sand | partial |
| Nakhon Nayok | 25 | Krok E Dok Waterfall | C | 14°27'34.3"N 101°11'45.09"E | 216 | sand | partial |
| Loei | 26 | Phu Ruea | NE | 17^o^30'51.6''N 101^o^20'43.1''E | 1,337 | rock | partial |
|  | 27 | Phu Kradueng | NE | 16°53'34.1"N 101°46'52.2"E | 1,215 | rock | partial |
| Nong Bua Lam Phu | 28 | Thao To Waterfall | NE | 17°13'51.5"N 102°27'44.6"E | 256 | sand | partial |
| Udon Thani | 29 | Than Ngam Waterfall | NE | 17°09'11.7"N 102°43'59.4"E | 288 | rock | open |
| Sakon Nakhon | 30 | Kham Hom Waterfall | NE | 17°07'25.0"N 104°10'10.2"E | 340 | rock | partial |
| Chaiyaphum | 31 | Theppana Waterfall | NE | 15°38'50.4"N 101°25'44.1"E | 576 | rock | open |
|  | 32 | Pa Hin Ngam | NE | 15°38'07.6"N 101°23'34.1"E | 712 | sand | open |

| **Province** | **Code** | **Names of sampling sites** | **Regions*** | **Latitude/Longitude** | **Elevation**  **(m)** | **Streambed**  **particle size** | **Canopy cover** |
| --- | --- | --- | --- | --- | --- | --- | --- |
| Mukdahan | 33 | Phu Hang | NE | 16°44'05.8"N 104°35'33.6"E | 161 | sand/rock | open |
| Ubon Ratchathani | 34 | Pha Taem | NE | 15°24'05.4"N 105°30'42.2"E | 200 | rock | open |
|  | 35 | Huai Yang | NE | 15°23'23.2"N 105°27'17.1"E | 110 | rock/sand | open |
| Sa Kaeo | 36 | Pang Sida Waterfall | E | 18°55'44.0"N 99°18'32.7"E | 127 | rock | partial |
| Prachinburi | 37 | Takhro Waterfall | E | 14^o^11'19.8''N 101^o^35'00.6''E | 76 | sand | partial |
| Rayong | 38 | Khao Chamao Waterfall | E | 12°54'55.0"N 101°43'51.3"E | 114 | rock | open |
| Chanthaburi | 39 | Phra Bat Pluang | E | 12°49'15.0"N 102°09'11.3"E | 191 | rock/sand | partial |
|  | 40 | Klong Piboon Stream | E | 12°53'09.5"N 102°06'51.4"E | 85 | sand/stone | open |
|  | 41 | Khao Soi Dao Waterfall | E | 13°05'03.6"N 102°10'19.9"E | 409 | rock | open |
| Trat | 42 | Klong Kaew Waterfall | E | 12^o^37'7.04"N 102^o^34'37.5"N | 315 | rock | partial |
| Tak | 43 | Doi Moozer | W | 16°45'35.6"N 98°55'31.3"E | 796 | sand | open |
|  | 44 | Nang Kruan Waterfall | W | 16°24'35.7"N 98°41'22.0"E | 402 | calcareous | partial |
|  | 45 | Khirirat Village | W | 16°27'24.2"N 98°54'29.5"E | 762 | calcareous | open |
| Kanchanaburi | 46 | Huai Mae Kamin Waterfall | W | 14^o^38'17.2''N 98^o^59'11.7''E | 256 | sand | partial |
| Ratchaburi | 47 | Phachondan Waterfall | W | 13^o^24'33.4"N 99^o^16'54.6"E | 205 | rock | open |
| Phetchaburi | 48 | Kaeng Krachan | W | 12^o^49'27.4''N 99^o^21'55.7''E | 953 | sand | partial |
|  | 49 | Sai Koo Waterfall | W | 11^o^14'43.9"N 99^o^20'52.2'"E | 111 | sand | open |

| **Province** | **Code** | **Names of sampling sites** | **Regions*** | **Latitude/Longitude** | **Elevation**  **(m)** | **Streambed**  **particle size** | **Canopy cover** |
| --- | --- | --- | --- | --- | --- | --- | --- |
| Prachuap Khiri Khan | 50 | Dong Mafai Waterfall | W | 12^o^03'07.9''N 99^o^36'47.1''E | 149 | rock | open |
| Chumphon | 51 | Klong Prao Waterfall | S | 10^o^02'25.3''N 98^o^57'55.9''E | 268 | sand | partial |
| Ranong | 52 | Namtok Ngao | S | 10^o^02'19.8''N 98^o^57'47.6''E | 286 | rock | partial |
| Surat Thani | 53 | Klong Mog Stream | S | 8^o^49'52.40''N 98^o^46'58.8''E | 80 | sand | partial |
| Krabi | 54 | Than Bok Khorani Waterfall | S | 8^o^20'49.70''N 98^o^43'49.4''E | 42 | rock | partial |
| Nakhon Si Thammarat | 55 | Nam Tok Yong | S | 8^o^10'20.91''N 99^o^44'42.81''E | 185 | sand | partial |
| Trang | 56 | Klong Krachong | S | 07°33'04.8"N 99°46' 90.2"E | 56 | stone | open |
| Phatthalung | 57 | Khao Pu-Khao Ya | S | 7^o^46'49.17''N 99^o^45'57.01''E | 560 | sand | partial |
| Songkhla | 58 | Khlong Ton Nga Chang | S | 06°56'51.1"N 100°15'15.7"E | 70 | stone | open |

* N = Northern; C = Central; NE = Northeastern; E = Eastern; W = Western; S = Southern
