# Supplementary material for: Biodiversity, seasonal abundance, and distribution of blackflies (Diptera: Simuliidae) in six different regions of Thailand
Source: Parasit Vectors. 2017 Nov 21;10:574. doi: 10.1186/s13071-017-2492-y (PMC5697434; doi:10.1186/s13071-017-2492-y)
Supplement: Supplementary file 2 — Diversity parameters for blackflies at 58 sampling sites in the six regions of Thailand. (DOCX 19 kb) [file 13071_2017_2492_MOESM2_ESM.docx]

**Additional file 2: Table S2.** Diversity parameters for blackflies at 58 sampling sites in the six regions of Thailand

| **Sampling site No.** | **Sampling sites** | | **Regions** | **Shannon_H** |
| --- | --- | --- | --- | --- |
| 1 | Namtok Mae Surin | | N | 1.9 |
| 2 | Huai Mae Sa | | N | 2 |
| 3 | Doi Chang | | N | 2 |
| 4 | Huai Kang Pla Waterfall | | N | 2 |
| 5 | Phu Kaeng Waterfall | | N | 1.5 |
| 6 | Phu Sang | | N | 2.2 |
| 7 | Doi Suthep | | N | 1.9 |
| 8 | Ban Khun Klang | | N | 1.9 |
| 9 | Doi Khun Tan | | N | 2 |
| 10 | Khun Tan service area | | N | 2.1 |
| 11 | Mae Wa | | N | 1.8 |
| 12 | Huai Rong Waterfall | | N | 1.2 |
| 13 | Doi Phu Kha | | N | 2.4 |
| 14 | Khun Sathan | | N | 2.1 |
| 15 | Phu Soi Dao Waterfall | | N | 2.3 |
| 16 | Tad Dean Waterfall | | C | 1.9 |
| 17 | Rom Klao | | C | 2.4 |
| 18 | Mae Ka Muennoi Waterfall | | C | 2.1 |
| 19 | Lan Hin Pum | | C | 1.7 |
| 20 | Klong Maepert | | C | 2 |
| 21 | Mae Wong | | C | 2.3 |
| 22 | Man Dang | | C | 2.2 |
| 23 | Huai Rabum | | C | 2.1 |
| 24 | Phu Toei | | C | 1.8 |
| 25 | Krok E Dok Waterfall | | C | 1.9 |
| 26 | Phu Ruea | | NE | 2.3 |
| 27 | Phu Kradueng | | NE | 2.1 |
| 28 | Thao To Waterfall | | NE | 1.8 |
| 29 | Than Ngam Waterfall | | NE | 2.1 |
| 30 | Kham Hom waterfall | | NE | 2.1 |
| 31 | Theppana Waterfall | | NE | 2.1 |
| **Sampling site No.** | | **Sampling sites** | **Regions** | **Shannon_H** |
| 32 | | Pha Hin Ngam | NE | 1.9 |
| 33 | | Phu Hang | NE | 2 |
| 34 | | Pha Team | NE | 1.8 |
| 35 | | Huai Yang | NE | 2.1 |
| 36 | | Pang Sida Waterfall | E | 1.7 |
| 37 | | Takhro Waterfall | E | 2.1 |
| 38 | | Khao Chamao Waterfall | E | 1.9 |
| 39 | | Phra Bat Pluang | E | 1.9 |
| 40 | | Klong Piboon Stream | E | 2 |
| 41 | | Khao Soi Dao Waterfall | E | 2 |
| 42 | | Klong Kaew Waterfall | E | 1.9 |
| 43 | | Doi Moozer | W | 2.2 |
| 44 | | Nang Kruan Waterfall | W | 1.3 |
| 45 | | Khirirat Village | W | 2.1 |
| 46 | | Huai Mae Kamin Waterfall | W | 2 |
| 47 | | Phachondan Waterfall | W | 2.1 |
| 48 | | Kaeng Krachan | W | 2 |
| 49 | | Sri Koo Waterfall | W | 1.9 |
| 50 | | Dong Mafai Waterfall | W | 2 |
| 51 | | Klong Prao Waterfall | S | 1.8 |
| 52 | | Namtok Ngao | S | 1.9 |
| 53 | | Klong Mog Stream | S | 1.9 |
| 54 | | Than Bok Khorani Waterfall | S | 1.7 |
| 55 | | Nam Tok Yong | S | 2.2 |
| 56 | | Klong Krachong | S | 1.4 |
| 57 | | Khao Pu-Khao Ya | S | 1.4 |
| 58 | | Khlong Ton Nga Chang | S | 1.5 |
