# Supplementary material for: Biodiversity, seasonal abundance, and distribution of blackflies (Diptera: Simuliidae) in six different regions of Thailand
Source: Parasit Vectors. 2017 Nov 21;10:574. doi: 10.1186/s13071-017-2492-y (PMC5697434; doi:10.1186/s13071-017-2492-y)
Supplement: Supplementary file 3 — Seasonal abundance and species richness of blackfly species at 58 sampling sites representing six regions in Thailand. (DOCX 44 kb) [file 13071_2017_2492_MOESM3_ESM.docx]

**Additional file 3: Table S3.** Seasonal abundance and species richness of blackfly species at 58 sampling sites representing six regions in Thailand

| **Species** | **Northern** | | | **Central** | | | **Northeastern** | | | **Eastern** | | | **Western** | | | **Southern** | | **Total** | **%flies** | **%SO** |
| --- | --- | --- | --- | --- | --- | --- | --- | --- | --- | --- | --- | --- | --- | --- | --- | --- | --- | --- | --- | --- |
|  | **R** | **C** | **H** | **R** | **C** | **H** | **R** | **C** | **H** | **R** | **C** | **H** | **R** | **C** | **H** | **R** | **H** |  |  |  |
| *Simulium* (*Asiosimulium*) *furvum* | 30 | 0 | 4 | 0 | 0 | 0 | 0 | 0 | 0 | 0 | 0 | 0 | 0 | 0 | 0 | 0 | 0 | 34 | 0.2 | 1.7 |
| *Simulium* (*Asiosimulium*) *oblongum* | 0 | 0 | 0 | 96 | 0 | 0 | 342 | 11 | 0 | 38 | 0 | 0 | 0 | 0 | 0 | 0 | 0 | 487 | 2.5 | 17.2 |
| *Simulium* (*Asiosimulium*) *wanchaii* | 24 | 0 | 2 | 0 | 0 | 0 | 8 | 0 | 0 | 0 | 0 | 0 | 0 | 0 | 0 | 0 | 0 | 34 | 0.2 | 3.5 |
| *Simulium* (*Daviesellum*) *pahangense* | 3 | 0 | 0 | 2 | 1 | 0 | 0 | 0 | 0 | 0 | 0 | 0 | 0 | 0 | 0 | 2 | 1 | 9 | 0.1 | 5.2 |
| *Simulium* (*Gomphostilbia*) *angulistylum* | 0 | 0 | 0 | 0 | 0 | 0 | 124 | 25 | 3 | 0 | 0 | 0 | 178 | 110 | 29 | 22 | 24 | 515 | 2.7 | 22.4 |
| *Simulium* (*Gomphostilbia*) *asakoae*#* | 142 | 200 | 47 | 202 | 131 | 20 | 276 | 118 | 21 | 96 | 6 | 9 | 80 | 128 | 8 | 64 | 60 | 1608 | 8.3 | 84.5 |
| *Simulium* (*Gomphostilbia*) *burtoni* | 0 | 13 | 6 | 10 | 13 | 1 | 6 | 0 | 0 | 0 | 0 | 0 | 0 | 0 | 0 | 146 | 328 | 523 | 2.7 | 24.1 |
| *Simulium* (*Gomphostilbia*) *chiangdaoense* | 79 | 182 | 37 | 58 | 104 | 55 | 0 | 0 | 0 | 0 | 0 | 0 | 0 | 0 | 0 | 0 | 0 | 515 | 2.7 | 10.3 |
| *Simulium* (*Gomphostilbia*) *chumpornense* | 0 | 3 | 0 | 5 | 13 | 0 | 51 | 19 | 13 | 6 | 5 | 0 | 26 | 12 | 1 | 9 | 19 | 182 | 0.9 | 27.6 |
| *Simulium* (*Gomphostilbia*) *curtatum* | 35 | 92 | 24 | 36 | 39 | 9 | 0 | 0 | 0 | 0 | 0 | 0 | 0 | 0 | 0 | 0 | 0 | 235 | 1.2 | 12.1 |
| *Simulium* (*Gomphostilbia*) *decuplum** | 131 | 193 | 34 | 131 | 48 | 39 | 119 | 65 | 2 | 156 | 178 | 35 | 34 | 8 | 2 | 0 | 0 | 1175 | 6 | 60.3 |
| *Simulium* (*Gomphostilbia*) *dentistylum* | 8 | 59 | 10 | 37 | 18 | 8 | 33 | 15 | 1 | 106 | 50 | 7 | 0 | 15 | 0 | 0 | 0 | 367 | 1.9 | 37.9 |
| *Simulium* (*Gomphostilbia*) *duolongum* | 0 | 0 | 0 | 91 | 22 | 20 | 62 | 0 | 0 | 0 | 0 | 0 | 176 | 56 | 23 | 0 | 0 | 450 | 2.3 | 22.4 |
| *Simulium* (*Gomphostilbia*) *gombakense* | 16 | 39 | 11 | 2 | 7 | 7 | 2 | 0 | 0 | 0 | 0 | 0 | 2 | 4 | 0 | 0 | 0 | 90 | 0.5 | 13.8 |
| *Simulium* (*Gomphostilbia*) *inthanonense* | 226 | 381 | 40 | 30 | 61 | 15 | 0 | 0 | 0 | 0 | 0 | 0 | 0 | 0 | 0 | 0 | 0 | 753 | 3.9 | 19 |
| *Simulium* (*Gomphostilbia*) *piroonae* | 21 | 8 | 23 | 0 | 0 | 0 | 0 | 0 | 0 | 0 | 0 | 0 | 0 | 0 | 0 | 0 | 0 | 52 | 0.3 | 1.72 |
| *Simulium* (*Gomphostilbia*) *kuvangkadilokae* | 0 | 0 | 0 | 0 | 0 | 0 | 61 | 32 | 5 | 0 | 0 | 0 | 0 | 0 | 0 | 0 | 0 | 98 | 0.5 | 3.5 |
| *Simulium* (*Gomphostilbia*) *parahiyangum* | 0 | 0 | 0 | 0 | 0 | 0 | 0 | 0 | 0 | 1 | 2 | 0 | 0 | 0 | 0 | 0 | 0 | 3 | 0 | 1.7 |
| *Simulium* (*Gomphostilbia*) *sheilae* | 2 | 20 | 0 | 9 | 5 | 1 | 36 | 8 | 0 | 16 | 2 | 2 | 110 | 37 | 19 | 134 | 155 | 556 | 2.9 | 44.8 |
| *Simulium* (*Gomphostilbia*) *siamense* complex **#* | 85 | 147 | 19 | 128 | 97 | 29 | 209 | 76 | 5 | 126 | 200 | 65 | 142 | 68 | 11 | 8 | 26 | 1441 | 7.4 | 75.9 |
| *Simulium* (*Montisimulium*) *nanense* | 15 | 34 | 2 | 0 | 0 | 0 | 0 | 0 | 0 | 0 | 0 | 0 | 0 | 0 | 0 | 0 | 0 | 51 | 0.3 | 1.7 |
| *Simulium* (*Montisimulium*) sp. | 0 | 0 | 0 | 17 | 31 | 9 | 0 | 0 | 0 | 0 | 0 | 0 | 0 | 0 | 0 | 0 | 0 | 57 | 0.3 | 1.7 |
| *Simulium* (*Nevermannia*) *aureohirtum* | 21 | 59 | 9 | 48 | 0 | 0 | 94 | 22 | 5 | 37 | 0 | 0 | 40 | 57 | 4 | 3 | 9 | 408 | 2.1 | 22.4 |
| **Species** | **Northern** | | | **Central** | | | **Northeastern** | | | **Eastern** | | | **Western** | | | **Southern** | | **Total** | **%flies** | **%SO** |
|  | **R** | **C** | **H** | **R** | **C** | **H** | **R** | **C** | **H** | **R** | **C** | **H** | **R** | **C** | **H** | **R** | **H** |  |  |  |
| *Simulium* (*Nevermannia*) *fangense* | 0 | 18 | 4 | 0 | 0 | 0 | 0 | 0 | 0 | 0 | 0 | 0 | 0 | 0 | 0 | 0 | 0 | 22 | 0.1 | 1.7 |
| *Simulium* (*Nevermannia*) *feuerborni* complex | 0 | 0 | 0 | 65 | 36 | 7 | 31 | 3 | 0 | 0 | 0 | 0 | 0 | 0 | 0 | 0 | 0 | 142 | 0.7 | 8.6 |
| *Simulium* (*Nevermannia*) *fruticosum* | 67 | 102 | 33 | 42 | 66 | 10 | 0 | 0 | 0 | 0 | 0 | 0 | 5 | 4 | 0 | 0 | 0 | 329 | 1.7 | 17.2 |
| *Simulium* (*Nevermannia*) *khunklangense* | 14 | 53 | 6 | 0 | 0 | 0 | 0 | 0 | 0 | 0 | 0 | 0 | 0 | 0 | 0 | 0 | 0 | 73 | 0.4 | 1.7 |
| *Simulium* (*Nevermannia*) *maeaiense* | 25 | 79 | 4 | 50 | 72 | 11 | 0 | 0 | 0 | 0 | 0 | 0 | 0 | 0 | 0 | 0 | 0 | 241 | 1.2 | 8.6 |
| *Simulium* (*Nevermannia*) *vessabutrae* | 1 | 6 | 0 | 0 | 0 | 0 | 0 | 0 | 0 | 0 | 0 | 0 | 0 | 0 | 0 | 0 | 0 | 7 | 0 | 1.7 |
| *Simulium* (*Simulium*) *atipornae* | 0 | 0 | 0 | 60 | 28 | 10 | 0 | 0 | 0 | 0 | 0 | 0 | 0 | 0 | 0 | 0 | 0 | 98 | 0.5 | 3.5 |
| *Simulium* (*Simulium*) *baimaii* | 0 | 0 | 0 | 0 | 0 | 0 | 50 | 19 | 0 | 0 | 0 | 0 | 0 | 0 | 0 | 0 | 0 | 69 | 0.4 | 1.7 |
| *Simulium* (*Simulium*) *brevipar* | 0 | 0 | 0 | 0 | 0 | 0 | 0 | 0 | 0 | 0 | 0 | 0 | 0 | 0 | 0 | 11 | 5 | 16 | 0.1 | 1.7 |
| *Simulium* (*Simulium*) *bullatum* | 8 | 19 | 11 | 11 | 0 | 0 | 0 | 0 | 0 | 0 | 0 | 0 | 8 | 4 | 0 | 0 | 0 | 61 | 0.3 | 10.3 |
| *Simulium* (*Simulium*) *chamlongi* | 42 | 87 | 16 | 28 | 17 | 12 | 0 | 0 | 0 | 0 | 0 | 0 | 10 | 5 | 0 | 0 | 0 | 217 | 1.1 | 20.7 |
| *Simulium* (*Simulium*) *chiangmaiense* | 30 | 44 | 0 | 0 | 0 | 0 | 0 | 0 | 0 | 0 | 0 | 0 | 0 | 5 | 0 | 0 | 0 | 79 | 0.4 | 3.5 |
| *Simulium (Simulium) doipuiense#* | 300 | 473 | 87 | 196 | 109 | 40 | 33 | 0 | 0 | 0 | 0 | 0 | 16 | 41 | 3 | 0 | 0 | 1298 | 6.7 | 25.9 |
| *Simulium* (*Simulium*) *fenestratum*#* | 152 | 181 | 25 | 105 | 68 | 19 | 216 | 108 | 14 | 216 | 190 | 73 | 149 | 36 | 33 | 36 | 60 | 1681 | 8.6 | 82.8 |
| *Simulium* (*Simulium*) *grossifilum* | 0 | 0 | 0 | 0 | 0 | 0 | 0 | 0 | 0 | 0 | 0 | 0 | 0 | 2 | 2 | 6 | 5 | 15 | 0.1 | 3.5 |
| *Simulium* (*Simulium*) *lampangense* | 41 | 52 | 9 | 0 | 0 | 0 | 0 | 0 | 0 | 0 | 0 | 0 | 10 | 27 | 0 | 0 | 0 | 139 | 0.7 | 5.2 |
| *Simulium* (*Simulium*) *lomkaoense* | 0 | 0 | 0 | 54 | 41 | 3 | 0 | 0 | 0 | 0 | 0 | 0 | 0 | 0 | 0 | 0 | 0 | 98 | 0.5 | 3.5 |
| *Simulium* (*Simulium*) *malayense* | 0 | 0 | 0 | 0 | 0 | 0 | 0 | 0 | 0 | 0 | 0 | 0 | 0 | 0 | 0 | 3 | 18 | 21 | 0.1 | 1.7 |
| *Simulium* (*Simulium*) *manooni* | 41 | 81 | 20 | 41 | 0 | 13 | 0 | 0 | 0 | 0 | 0 | 0 | 0 | 0 | 0 | 0 | 0 | 196 | 1 | 8.6 |
| *Simulium* (*Simulium*) *nakhonense*#* | 67 | 78 | 24 | 148 | 87 | 16 | 42 | 4 | 0 | 115 | 21 | 4 | 381 | 110 | 57 | 102 | 195 | 1451 | 7.5 | 58.6 |
| *Simulium* (*Simulium*) *nigrogilvum* | 0 | 6 | 1 | 6 | 4 | 0 | 0 | 0 | 0 | 0 | 0 | 0 | 0 | 0 | 0 | 0 | 0 | 17 | 0.1 | 5.2 |
| *Simulium* (*Simulium*) *nobile* | 0 | 0 | 0 | 0 | 0 | 0 | 0 | 0 | 0 | 0 | 0 | 0 | 67 | 24 | 6 | 203 | 543 | 843 | 4.3 | 17.2 |
| *Simulium* (*Simulium*) *nodosum* | 54 | 39 | 9 | 2 | 11 | 0 | 0 | 0 | 0 | 0 | 0 | 0 | 119 | 41 | 26 | 0 | 0 | 301 | 1.5 | 15.5 |
| *Simulium* (*Simulium*) *phayaoense* | 0 | 11 | 0 | 0 | 0 | 0 | 0 | 0 | 0 | 32 | 9 | 2 | 0 | 0 | 0 | 0 | 0 | 54 | 0.3 | 6.9 |
| *Simulium* (*Simulium*) *prayongi* | 0 | 0 | 0 | 0 | 0 | 0 | 0 | 0 | 0 | 0 | 0 | 0 | 10 | 21 | 4 | 0 | 0 | 35 | 0.2 | 1.7 |
| **Species** | **Northern** | | | **Central** | | | **Northeastern** | | | **Eastern** | | | **Western** | | | **Southern** | | **Total** | **%flies** | **%SO** |
|  | **R** | **C** | **H** | **R** | **C** | **H** | **R** | **C** | **H** | **R** | **C** | **H** | **R** | **C** | **H** | **R** | **H** |  |  |  |
| *Simulium* (*Simulium*) *phukaense* | 10 | 7 | 0 | 20 | 0 | 0 | 0 | 0 | 0 | 0 | 0 | 0 | 0 | 0 | 0 | 0 | 0 | 37 | 0.2 | 6.9 |
| *Simulium* (*Simulium*) *quinquestriatum* | 33 | 43 | 8 | 74 | 55 | 22 | 150 | 58 | 11 | 31 | 24 | 3 | 35 | 67 | 0 | 0 | 0 | 614 | 3.2 | 34.1 |
| *Simulium* (*Simulium*) *siripoomense* | 0 | 17 | 2 | 0 | 0 | 0 | 0 | 0 | 0 | 0 | 0 | 0 | 0 | 0 | 0 | 0 | 0 | 19 | 0.1 | 1.7 |
| *Simulium* (*Simulium*) *takense* | 0 | 0 | 0 | 0 | 0 | 0 | 0 | 0 | 0 | 0 | 0 | 0 | 32 | 27 | 10 | 0 | 0 | 69 | 0.4 | 1.7 |
| *Simulium* (*Simulium*) *tani* complex*** | 5 | 30 | 4 | 9 | 0 | 0 | 14 | 7 | 0 | 75 | 51 | 6 | 72 | 16 | 9 | 51 | 36 | 385 | 2 | 48.3 |
| *Simulium* (*Simulium*) *thailandicum* | 39 | 40 | 4 | 0 | 0 | 0 | 0 | 0 | 0 | 0 | 0 | 0 | 57 | 12 | 0 | 44 | 32 | 228 | 1.2 | 12.1 |
| *Simulium* (*Simulium*) *weji* | 87 | 106 | 10 | 0 | 0 | 0 | 0 | 0 | 0 | 0 | 0 | 0 | 198 | 147 | 61 | 0 | 0 | 609 | 3.1 | 6.9 |
| *Simulium* (*Simulium*) *yongi* | 0 | 0 | 0 | 0 | 2 | 0 | 0 | 0 | 0 | 0 | 0 | 0 | 0 | 0 | 0 | 18 | 18 | 38 | 0.2 | 3.5 |
| *Simulium* (*Simulium*) *yuphae* | 78 | 88 | 21 | 29 | 22 | 14 | 2 | 4 | 2 | 5 | 0 | 0 | 34 | 8 | 4 | 0 | 0 | 311 | 1.6 | 36.2 |
| **Total** | **1932** | **3090** | **566** | **1842** | **1208** | **390** | **1961** | **594** | **82** | **1056** | **738** | **206** | **1991** | **1092** | **312** | **862** | **1534** | **19,456** | **100.00** |  |
| **Seasonal species richness** | **34** | **37** | **33** | **33** | **28** | **24** | **22** | **17** | **11** | **15** | **12** | **10** | **25** | **28** | **19** | **17** | **17** |  |  |  |
| **Regional species richness** | **40** | | | **34** | | | **22** | | | **15** | | | **28** | | | **17** | |  |  |  |

* The most frequent taxa at all sites

# The most predominant taxa
