# Supplementary material for: Biodiversity, seasonal abundance, and distribution of blackflies (Diptera: Simuliidae) in six different regions of Thailand
Source: Parasit Vectors. 2017 Nov 21;10:574. doi: 10.1186/s13071-017-2492-y (PMC5697434; doi:10.1186/s13071-017-2492-y)
Supplement: Supplementary file 4 — Regional distribution and relative abundance of blackflies at 15 sampling sites in northern Thailand. (DOCX 30 kb) [file 13071_2017_2492_MOESM4_ESM.docx]

**Additional file 4: Table S4.** Regional distribution and relative abundance of blackflies at 15 sampling sites in northern Thailand

| **Species** | **Sampling site No.** | | | | | | | | | | | | | | | **Total** | **%flies** | **%SO** |
| --- | --- | --- | --- | --- | --- | --- | --- | --- | --- | --- | --- | --- | --- | --- | --- | --- | --- | --- |
|  | **1** | **2** | **3** | **4** | **5** | **6** | **7** | **8** | **9** | **10** | **11** | **12** | **13** | **14** | **15** |  |  |  |
| *S.* (*A.*) *furvum* | 0 | 0 | 0 | 0 | 0 | 0 | 0 | 0 | 0 | 0 | 34 | 0 | 0 | 0 | 0 | 34 | 0.6 | 6.7 |
| *S.*(*A.*) *wanchaii* | 0 | 0 | 0 | 0 | 0 | 0 | 0 | 0 | 0 | 0 | 26 | 0 | 0 | 0 | 0 | 26 | 0.5 | 6.7 |
| *S.*(*D.*) *pahangense* | 0 | 0 | 0 | 0 | 0 | 0 | 0 | 0 | 0 | 0 | 0 | 0 | 3 | 0 | 0 | 3 | 0.1 | 6.7 |
| *S.*(*G.*) *asakoae* complex | 37 | 10 | 42 | 54 | 0 | 16 | 20 | 0 | 88 | 40 | 24 | 0 | 22 | 29 | 7 | 389 | 7 | 80 |
| *S.*(*G*.) *burtoni* | 0 | 0 | 0 | 19 | 0 | 0 | 0 | 0 | 0 | 0 | 0 | 0 | 0 | 0 | 0 | 19 | 0.3 | 6.7 |
| *S.*(*G.*) *chiangdaoense* | 0 | 0 | 0 | 0 | 0 | 0 | 0 | 155 | 0 | 0 | 0 | 0 | 98 | 45 | 0 | 298 | 5.3 | 20 |
| *S.*(*G.*) *chumpornense* | 0 | 3 | 0 | 0 | 0 | 0 | 0 | 0 | 0 | 0 | 0 | 0 | 0 | 0 | 0 | 3 | 0.1 | 6.7 |
| *S.*(*G.*) *curtatum* | 0 | 0 | 2 | 0 | 0 | 18 | 66 | 41 | 0 | 0 | 0 | 0 | 24 | 0 | 0 | 151 | 2.7 | 33.3 |
| *S.*(*G.*) *decuplum* | 31 | 0 | 78 | 129 | 0 | 49 | 0 | 3 | 0 | 38 | 0 | 0 | 0 | 8 | 22 | 358 | 6.4 | 53.3 |
| *S.*(*G.*) *dentistylum* | 0 | 0 | 13 | 0 | 0 | 0 | 7 | 0 | 0 | 40 | 0 | 0 | 0 | 0 | 17 | 77 | 1.4 | 26.7 |
| *S.*(*G.*) *gombakense* | 0 | 0 | 0 | 0 | 0 | 0 | 9 | 0 | 23 | 0 | 16 | 0 | 9 | 9 | 0 | 66 | 1.2 | 33.3 |
| *S.*(*G.*) *inthanonense* | 31 | 0 | 86 | 0 | 0 | 28 | 68 | 214 | 82 | 0 | 0 | 0 | 59 | 79 | 0 | 647 | 11.6 | 53.3 |
| *S.*(*G.*) *piroonae* | 0 | 52 | 0 | 0 | 0 | 0 | 0 | 0 | 0 | 0 | 0 | 0 | 0 | 0 | 0 | 52 | 0.9 | 6.7 |
| *S.*(*G.*) *sheilae* | 0 | 0 | 0 | 5 | 0 | 0 | 4 | 0 | 0 | 11 | 0 | 0 | 0 | 0 | 2 | 22 | 0.4 | 26.7 |
| *S.*(*G.*) *siamense* complex | 0 | 76 | 0 | 0 | 11 | 37 | 18 | 0 | 31 | 40 | 0 | 4 | 3 | 11 | 20 | 251 | 4.5 | 66.7 |
| *S.*(*M.*) *nanense* | 0 | 0 | 0 | 0 | 0 | 0 | 0 | 0 | 0 | 0 | 0 | 0 | 51 | 0 | 0 | 51 | 0.9 | 6.7 |
| *S.*(*N.*) *aureohirtum* | 0 | 0 | 0 | 0 | 0 | 0 | 0 | 0 | 10 | 48 | 31 | 0 | 0 | 0 | 0 | 89 | 1.6 | 20 |
| *S.*(*N.*) *fangense* | 0 | 0 | 0 | 0 | 0 | 0 | 0 | 0 | 0 | 0 | 0 | 0 | 22 | 0 | 0 | 22 | 0.4 | 6.7 |
| *S.*(*N.*) *fruticosum* | 0 | 0 | 0 | 0 | 0 | 4 | 11 | 9 | 51 | 0 | 0 | 0 | 44 | 83 | 0 | 202 | 3.6 | 40 |
| *S.*(*N.*) *khunklangense* | 0 | 0 | 0 | 0 | 0 | 0 | 0 | 73 | 0 | 0 | 0 | 0 | 0 | 0 | 0 | 73 | 1.3 | 6.7 |
| *S.*(*N.*) *maeaiense* | 0 | 0 | 0 | 0 | 0 | 0 | 0 | 36 | 0 | 0 | 0 | 0 | 70 | 2 | 0 | 108 | 1.9 | 20 |
|  |  | | | | | | | | | | | | | | |  |  |  |
|  |  | | | | | | | | | | | | | | |  |  |  |
| **Species** | **Sampling sites No.** | | | | | | | | | | | | | | | **Total** | **%flies** | **%SO** |
|  | **1** | **2** | **3** | **4** | **5** | **6** | **7** | **8** | **9** | **10** | **11** | **12** | **13** | **14** | **15** |  |  |  |
| *S.*(*N.*) *vessabutrae* | 0 | 0 | 0 | 0 | 0 | 0 | 0 | 0 | 0 | 0 | 0 | 0 | 0 | 7 | 0 | 7 | 0.1 | 6.7 |
| *S.*(*S.*) *bullatum* | 13 | 0 | 0 | 0 | 0 | 0 | 0 | 0 | 0 | 0 | 0 | 0 | 3 | 8 | 14 | 38 | 0.7 | 26.7 |
| *S.*(*S.*) *chamlongi* | 35 | 0 | 15 | 0 | 0 | 8 | 4 | 49 | 26 | 0 | 0 | 0 | 8 | 0 | 0 | 145 | 2.6 | 46.7 |
| *S.*(*S.*) *chiangmaiense* | 0 | 74 | 0 | 0 | 0 | 0 | 0 | 0 | 0 | 0 | 0 | 0 | 0 | 0 | 0 | 74 | 1.3 | 6.7 |
| *S.*(*S.*) *doipuiense* complex | 168 | 0 | 104 | 0 | 0 | 0 | 149 | 150 | 115 | 0 | 0 | 0 | 110 | 64 | 0 | 860 | 15.4 | 46.7 |
| *S.*(*S.*) *fenestratum* | 34 | 12 | 47 | 20 | 38 | 40 | 0 | 0 | 8 | 40 | 9 | 37 | 0 | 11 | 62 | 358 | 6.4 | 80 |
| *S.*(*S.*) *lampangense* | 0 | 0 | 0 | 0 | 61 | 0 | 0 | 0 | 0 | 0 | 0 | 41 | 0 | 0 | 0 | 102 | 1.8 | 13.3 |
| *S.*(*S.*) *manooni* | 81 | 0 | 0 | 0 | 0 | 0 | 33 | 0 | 0 | 0 | 0 | 0 | 28 | 0 | 0 | 142 | 2.5 | 20 |
| *S.*(*S.*) *nakhonense* | 0 | 27 | 0 | 92 | 0 | 28 | 0 | 0 | 0 | 6 | 0 | 0 | 0 | 0 | 16 | 169 | 3 | 33.3 |
| *S.*(*S.*) *nigrogilvum* | 2 | 0 | 0 | 0 | 0 | 0 | 0 | 0 | 0 | 0 | 0 | 0 | 0 | 0 | 5 | 7 | 0.1 | 13.3 |
| *S.*(*S.*) *nodosum* | 0 | 19 | 27 | 53 | 0 | 0 | 0 | 0 | 0 | 0 | 0 | 3 | 0 | 0 | 0 | 102 | 1.8 | 26.7 |
| *S.*(*S.*) *phayaoense* | 0 | 0 | 0 | 0 | 0 | 0 | 0 | 0 | 0 | 0 | 0 | 0 | 0 | 0 | 11 | 11 | 0.2 | 6.7 |
| *S.*(*S.*) *phukaense* | 0 | 0 | 0 | 0 | 14 | 0 | 0 | 0 | 0 | 0 | 0 | 0 | 2 | 1 | 0 | 17 | 0.3 | 20 |
| *S.*(*S.*) *quinquestriatum* | 0 | 0 | 0 | 19 | 26 | 2 | 0 | 0 | 0 | 0 | 0 | 0 | 0 | 0 | 37 | 84 | 1.5 | 26.7 |
| *S.*(*S.*) *siripoomense* | 0 | 19 | 0 | 0 | 0 | 0 | 0 | 0 | 0 | 0 | 0 | 0 | 0 | 0 | 0 | 19 | 0.3 | 6.7 |
| *S.*(*S.*) *tani* complex | 12 | 0 | 0 | 6 | 0 | 0 | 0 | 0 | 0 | 8 | 0 | 0 | 0 | 0 | 13 | 39 | 0.7 | 26.7 |
| *S.*(*S.*) *thailandicum* | 0 | 55 | 0 | 28 | 0 | 0 | 0 | 0 | 0 | 0 | 0 | 0 | 0 | 0 | 0 | 83 | 1.5 | 13.3 |
| *S.*(*S.*) *weji* | 0 | 0 | 0 | 0 | 109 | 0 | 0 | 0 | 0 | 0 | 0 | 94 | 0 | 0 | 0 | 203 | 3.6 | 13.3 |
| *S.*(*S.*) *yuphae* | 0 | 0 | 25 | 10 | 0 | 14 | 24 | 32 | 27 | 5 | 8 | 0 | 20 | 15 | 7 | 187 | 3.4 | 73.3 |
| **Total** | **444** | **347** | **439** | **435** | **259** | **244** | **413** | **762** | **461** | **276** | **148** | **179** | **576** | **372** | **233** | **5,588** | **100** |  |
