# Supplementary material for: Biodiversity, seasonal abundance, and distribution of blackflies (Diptera: Simuliidae) in six different regions of Thailand
Source: Parasit Vectors. 2017 Nov 21;10:574. doi: 10.1186/s13071-017-2492-y (PMC5697434; doi:10.1186/s13071-017-2492-y)
Supplement: Supplementary file 5 — Regional distribution and relative abundance of blackflies at 10 sampling sites in central Thailand. (DOCX 24 kb) [file 13071_2017_2492_MOESM5_ESM.docx]

**Additional file 5: Table S5.** Regional distribution and relative abundance of blackflies at 10 sampling sites in central Thailand

| **Species** | **Sampling site No.** | | | | | | | | | | | | |
| --- | --- | --- | --- | --- | --- | --- | --- | --- | --- | --- | --- | --- | --- |
|  | **16** | **17** | **18** | **19** | **20** | **21** | **22** | **23** | **24** | **25** | **Total** | **%flies** | **%SO** |
| *S.* (*A.*) *oblongum* | 0 | 0 | 0 | 96 | 0 | 0 | 0 | 0 | 0 | 0 | 96 | 2.8 | 10 |
| *S.* (*D.*) *pahangense* | 0 | 0 | 0 | 0 | 0 | 3 | 0 | 0 | 0 | 0 | 3 | 0.1 | 10 |
| *S.* (*G.*) *asakoae* complex | 17 | 50 | 0 | 16 | 17 | 146 | 4 | 11 | 47 | 45 | 353 | 10.3 | 90 |
| *S.* (*G.*) *burtoni* | 0 | 0 | 0 | 0 | 5 | 0 | 0 | 6 | 0 | 13 | 24 | 0.7 | 30 |
| *S.* (*G.*) *chiangdaoense* | 0 | 74 | 0 | 0 | 0 | 96 | 47 | 0 | 0 | 0 | 217 | 6.3 | 30 |
| *S.* (*G.*) *chumpornense* | 0 | 0 | 0 | 0 | 0 | 0 | 0 | 18 | 0 | 0 | 18 | 0.5 | 10 |
| *S.* (*G.*) *curtatum* | 0 | 0 | 0 | 0 | 0 | 52 | 32 | 0 | 0 | 0 | 84 | 2.4 | 20 |
| *S.* (*G.*) *decuplum* | 44 | 9 | 44 | 0 | 19 | 0 | 8 | 29 | 48 | 17 | 218 | 6.3 | 80 |
| *S.* (*G.*) *dentistylum* | 30 | 3 | 0 | 0 | 0 | 0 | 0 | 10 | 7 | 13 | 63 | 1.8 | 50 |
| *S.* (*G.*) *duolongum* | 0 | 0 | 0 | 0 | 46 | 0 | 0 | 77 | 10 | 0 | 133 | 3.9 | 30 |
| *S.* (*G.*) *gombakense* | 0 | 16 | 0 | 0 | 0 | 0 | 0 | 0 | 0 | 0 | 16 | 0.5 | 10 |
| *S.* (*G.*) *inthanonense* | 0 | 19 | 0 | 0 | 0 | 42 | 45 | 0 | 0 | 0 | 106 | 3.1 | 30 |
| *S.* (*G.*) *sheilae* | 0 | 0 | 0 | 0 | 0 | 0 | 6 | 0 | 7 | 2 | 15 | 0.4 | 30 |
| *S.* (*G.*) *siamense* complex | 30 | 16 | 33 | 17 | 20 | 10 | 0 | 39 | 47 | 42 | 254 | 7.4 | 90 |
| *S.* (*M.*) sp. | 0 | 0 | 0 | 0 | 0 | 57 | 0 | 0 | 0 | 0 | 57 | 1.7 | 10 |
| *S.* (*N.*) *aureohirtum* | 0 | 0 | 21 | 27 | 0 | 0 | 0 | 0 | 0 | 0 | 48 | 1.4 | 20 |
| *S.* (*N.*) *feuerborni* complex | 0 | 10 | 17 | 81 | 0 | 0 | 0 | 0 | 0 | 0 | 108 | 3.1 | 30 |
| *S.* (*N.*) *fruticosum* | 0 | 63 | 0 | 0 | 0 | 36 | 19 | 0 | 0 | 0 | 118 | 3.4 | 30 |
| *S.* (*N.*) *maeaiense* | 0 | 82 | 0 | 0 | 0 | 51 | 0 | 0 | 0 | 0 | 133 | 3.9 | 20 |
| *S.* (*S.*) *atipornae* | 0 | 0 | 40 | 0 | 0 | 0 | 58 | 0 | 0 | 0 | 98 | 2.9 | 20 |
| *S.* (*S.*) *bullatum* | 0 | 11 | 0 | 0 | 0 | 0 | 0 | 0 | 0 | 0 | 11 | 0.3 | 10 |
| *S.* (*S.*) *chamlongi* | 0 | 0 | 30 | 0 | 0 | 4 | 15 | 0 | 8 | 0 | 57 | 1.7 | 40 |
| **species** | **Sampling sites No.** | | | | | | | | | | | | |
|  | **16** | **17** | **18** | **19** | **20** | **21** | **22** | **23** | **24** | **25** | **Total** | **%flies** | **%SO** |
| *S.* (*S.*) *doipuiense* complex | 0 | 89 | 98 | 28 | 0 | 56 | 74 | 0 | 0 | 0 | 345 | 10 | 50 |
| *S.* (*S.*) *fenestratum* | 47 | 3 | 13 | 0 | 25 | 0 | 20 | 14 | 7 | 63 | 192 | 5.6 | 80 |
| *S.* (*S.*) *lomkaoense* | 0 | 0 | 34 | 0 | 0 | 0 | 64 | 0 | 0 | 0 | 98 | 2.9 | 20 |
| *S.* (*S.*) *manooni* | 0 | 32 | 0 | 0 | 0 | 22 | 0 | 0 | 0 | 0 | 54 | 1.6 | 20 |
| *S.* (*S.*) *nakhonense* | 26 | 0 | 0 | 0 | 74 | 0 | 0 | 56 | 85 | 10 | 251 | 7.3 | 50 |
| *S.* (*S.*) *nigrogilvum* | 0 | 0 | 0 | 0 | 0 | 10 | 0 | 0 | 0 | 0 | 10 | 0.3 | 10 |
| *S.* (*S.*) *nodosum* | 0 | 0 | 0 | 0 | 13 | 0 | 0 | 0 | 0 | 0 | 13 | 0.4 | 10 |
| *S.* (*S.*) *phukaense* | 0 | 20 | 0 | 0 | 0 | 0 | 0 | 0 | 0 | 0 | 20 | 0.6 | 10 |
| *S.* (*S.*) *quinquestriatum* | 51 | 0 | 0 | 0 | 46 | 0 | 0 | 16 | 0 | 38 | 151 | 4.4 | 40 |
| *S.* (*S.*) *tani* complex | 0 | 0 | 0 | 0 | 0 | 0 | 0 | 9 | 0 | 0 | 9 | 0.3 | 10 |
| *S.* (*S.*) *yongi* | 0 | 0 | 0 | 0 | 0 | 2 | 0 | 0 | 0 | 0 | 2 | 0.1 | 10 |
| *S.* (*S.*) *yuphae* | 0 | 23 | 14 | 18 | 0 | 10 | 0 | 0 | 0 | 0 | 65 | 1.9 | 40 |
| **Total** | **245** | **520** | **344** | **283** | **265** | **597** | **392** | **285** | **266** | **243** | **3440** | **100** |  |
