# Supplementary material for: Biodiversity, seasonal abundance, and distribution of blackflies (Diptera: Simuliidae) in six different regions of Thailand
Source: Parasit Vectors. 2017 Nov 21;10:574. doi: 10.1186/s13071-017-2492-y (PMC5697434; doi:10.1186/s13071-017-2492-y)
Supplement: Supplementary file 6 — Regional distribution and relative abundance of blackflies at 10 sampling sites in northeastern Thailand. (DOCX 20 kb) [file 13071_2017_2492_MOESM6_ESM.docx]

**Additional file 6: Table S6.** Regional distribution and relative abundance of blackflies at 10 sampling sites in northeastern Thailand

| **Species** | **Sampling site No.** | | | | | | | | | | | | |
| --- | --- | --- | --- | --- | --- | --- | --- | --- | --- | --- | --- | --- | --- |
|  | **26** | **27** | **28** | **29** | **30** | **31** | **32** | **33** | **34** | **35** | **Total** | **%flies** | **%SO** |
| *S.* (*A.*) *oblongum* | 18 | 24 | 0 | 66 | 32 | 45 | 41 | 62 | 65 | 0 | 353 | 13.4 | 80 |
| *S.* (*A.*) *wanchaii* | 8 | 0 | 0 | 0 | 0 | 0 | 0 | 0 | 0 | 0 | 8 | 0.3 | 10 |
| *S.* (*G.*) *angulistylum* complex | 0 | 0 | 0 | 0 | 60 | 50 | 0 | 42 | 0 | 0 | 152 | 5.8 | 30 |
| *S.* (*G.*) *asakoae* complex | 52 | 32 | 45 | 79 | 55 | 31 | 2 | 19 | 53 | 47 | 415 | 15.7 | 100 |
| *S.* (*G.*) *burtoni* | 1 | 0 | 0 | 5 | 0 | 0 | 0 | 0 | 0 | 0 | 6 | 0.2 | 20 |
| *S.* (*G.*) *chumpornense* | 44 | 13 | 2 | 0 | 11 | 5 | 8 | 0 | 0 | 0 | 83 | 3.2 | 60 |
| *S.* (*G.*) *decuplum* | 3 | 22 | 31 | 62 | 9 | 12 | 16 | 0 | 12 | 19 | 186 | 7.1 | 90 |
| *S.* (*G.*) *dentistylum* | 0 | 21 | 7 | 7 | 0 | 0 | 0 | 0 | 4 | 10 | 49 | 1.9 | 50 |
| *S.* (*G.*) *duolongum* | 0 | 0 | 7 | 0 | 6 | 13 | 1 | 21 | 0 | 14 | 62 | 2.4 | 60 |
| *S.* (*G.*) *gombakense* | 0 | 0 | 0 | 2 | 0 | 0 | 0 | 0 | 0 | 0 | 2 | 0.1 | 10 |
| *S.* (*G.*) *kuvangkadilokae* | 0 | 0 | 0 | 0 | 0 | 0 | 0 | 40 | 0 | 58 | 98 | 3.7 | 20 |
| *S.* (*G.*) *sheilae* | 0 | 0 | 0 | 11 | 12 | 0 | 21 | 0 | 0 | 0 | 44 | 1.7 | 30 |
| *S.* (*G.*) *siamense* complex | 21 | 4 | 28 | 85 | 27 | 22 | 29 | 26 | 25 | 23 | 290 | 11 | 100 |
| *S.* (*G.*) *aureohirtum* | 33 | 6 | 0 | 0 | 0 | 0 | 69 | 0 | 13 | 0 | 121 | 4.6 | 40 |
| *S.* (*G.*) *feuerborni* complex | 22 | 12 | 0 | 0 | 0 | 0 | 0 | 0 | 0 | 0 | 34 | 1.3 | 20 |
| *S.* (*S.*) *baimaii* | 0 | 69 | 0 | 0 | 0 | 0 | 0 | 0 | 0 | 0 | 69 | 2.6 | 10 |
| *S.* (*S.*) *doipuiense* complex | 33 | 0 | 0 | 0 | 0 | 0 | 0 | 0 | 0 | 0 | 33 | 1.3 | 10 |
| *S.* (*S.*) *fenestratum* | 54 | 38 | 30 | 71 | 38 | 52 | 3 | 21 | 0 | 31 | 338 | 12.8 | 90 |
| *S.* (*S.*) *nakhonense* | 0 | 0 | 0 | 15 | 10 | 0 | 0 | 0 | 9 | 12 | 46 | 1.7 | 40 |
| *S.* (*S.*) *quinquestriatum* | 25 | 0 | 17 | 28 | 12 | 35 | 19 | 12 | 14 | 57 | 219 | 8.3 | 90 |
| *S.* (*S.*) *tani* complex | 2 | 3 | 0 | 9 | 0 | 2 | 0 | 0 | 2 | 3 | 21 | 0.8 | 60 |
| *S.* (*S.*) *yuphae* | 8 | 0 | 0 | 0 | 0 | 0 | 0 | 0 | 0 | 0 | 8 | 0.3 | 10 |
| **Total** | **324** | **244** | **167** | **440** | **272** | **267** | **209** | **243** | **197** | **274** | **2,637** | **100.00** |  |
