# Supplementary material for: Biodiversity, seasonal abundance, and distribution of blackflies (Diptera: Simuliidae) in six different regions of Thailand
Source: Parasit Vectors. 2017 Nov 21;10:574. doi: 10.1186/s13071-017-2492-y (PMC5697434; doi:10.1186/s13071-017-2492-y)
Supplement: Supplementary file 7 — Regional distribution and relative abundance of blackflies at 7 sampling sites in eastern Thailand. (DOCX 18 kb) [file 13071_2017_2492_MOESM7_ESM.docx]

**Additional file 7: Table S7.** Regional distribution and relative abundance of blackflies at 7 sampling sites in eastern Thailand

| **Species** | **Sampling site No.** | | | | | | | | | |
| --- | --- | --- | --- | --- | --- | --- | --- | --- | --- | --- |
|  | **36** | **37** | **38** | **39** | **40** | **41** | **42** | **Total** | **%flies** | **%SO** |
| *S.* (*A.*) *oblongum* | 38 | 0 | 0 | 0 | 0 | 0 | 0 | 38 | 1.9 | 14.3 |
| *S.* (*G.*) *asakoae* complex | 0 | 18 | 24 | 13 | 11 | 18 | 27 | 111 | 5.6 | 85.7 |
| *S.* (*G.*) *chumpornense* | 0 | 11 | 0 | 0 | 0 | 0 | 0 | 11 | 0.6 | 14.3 |
| *S.* (*G.*) *decuplum* | 14 | 64 | 56 | 54 | 68 | 69 | 44 | 369 | 18.5 | 100 |
| *S.* (*G.*) *dentistylum* | 35 | 15 | 4 | 28 | 46 | 22 | 13 | 163 | 8.2 | 100 |
| *S.* (*G.*) *parahiyangum* | 0 | 3 | 0 | 0 | 0 | 0 | 0 | 3 | 0.2 | 14.3 |
| *S.* (*G.*) *sheilae* | 0 | 6 | 10 | 0 | 4 | 0 | 0 | 20 | 1 | 42.9 |
| *S.* (*G*.) *siamense* complex | 75 | 54 | 46 | 49 | 65 | 44 | 58 | 391 | 19.6 | 100 |
| *S.* (*N.*) *aureohirtum* | 0 | 0 | 0 | 0 | 0 | 37 | 0 | 37 | 1.9 | 14.3 |
| *S.* (*S.*) *fenestratum* | 65 | 67 | 51 | 60 | 83 | 77 | 76 | 479 | 24 | 100 |
| *S*. (*S*.) *nakhonense* | 0 | 19 | 22 | 45 | 28 | 8 | 18 | 140 | 7 | 85.7 |
| *S.* (*S.*) *phayaoense* | 0 | 0 | 0 | 22 | 0 | 14 | 7 | 43 | 2.2 | 42.9 |
| *S.* (*S.*) *quinquestriatum* | 17 | 20 | 4 | 0 | 17 | 0 | 0 | 58 | 2.9 | 57.1 |
| *S.* (*S*.) *tani* complex | 5 | 19 | 11 | 12 | 45 | 17 | 23 | 132 | 6.6 | 100 |
| *S.* (*S*.) *yuphae* | 0 | 0 | 1 | 0 | 4 | 0 | 0 | 5 | 0.3 | 28.6 |
| **Total** | **249** | **296** | **229** | **283** | **371** | **306** | **266** | **2000** | **100.00** |  |
