# Supplementary material for: Biodiversity, seasonal abundance, and distribution of blackflies (Diptera: Simuliidae) in six different regions of Thailand
Source: Parasit Vectors. 2017 Nov 21;10:574. doi: 10.1186/s13071-017-2492-y (PMC5697434; doi:10.1186/s13071-017-2492-y)
Supplement: Supplementary file 8 — Regional distribution and relative abundance of blackflies at 8 sampling sites in western Thailand. (DOCX 21 kb) [file 13071_2017_2492_MOESM8_ESM.docx]

**Additional file 8: Table S8.** Regional distribution and relative abundance of blackflies at 8 sampling sites in western Thailand

| **Species** | **Sampling site No.** | | | | | | | | | | |
| --- | --- | --- | --- | --- | --- | --- | --- | --- | --- | --- | --- |
|  | **43** | **44** | **45** | **46** | **47** | **48** | **49** | **50** | **Total** | **%flies** | **%SO** |
| *S.* (*G.*) *angulistylum* complex | 0 | 0 | 0 | 26 | 70 | 61 | 116 | 44 | 317 | 9.3 | 62.5 |
| *S.* (*G.*) *asakoae* complex | 75 | 0 | 38 | 35 | 27 | 41 | 0 | 0 | 216 | 6.4 | 62.5 |
| *S.* (*G.*) *chumpornense* | 0 | 0 | 0 | 0 | 5 | 4 | 22 | 8 | 39 | 1.2 | 50 |
| *S.* (*G.*) *decuplum* | 14 | 0 | 13 | 17 | 0 | 0 | 0 | 0 | 44 | 1.3 | 37.5 |
| *S.* (*G.*) *dentistylum* | 0 | 0 | 0 | 0 | 15 | 0 | 0 | 0 | 15 | 0.4 | 12.5 |
| *S.* (*G.*) *duolongum* | 0 | 0 | 0 | 0 | 43 | 80 | 99 | 33 | 255 | 7.5 | 50 |
| *S.* (*G.*) *gombakense* | 0 | 0 | 6 | 0 | 0 | 0 | 0 | 0 | 6 | 0.2 | 12.5 |
| *S.* (*G.*) *sheilae* | 4 | 0 | 0 | 0 | 23 | 20 | 85 | 34 | 166 | 4.9 | 62.5 |
| *S.* (*G.*) *siamense* complex | 0 | 0 | 0 | 91 | 21 | 34 | 13 | 62 | 221 | 6.5 | 62.5 |
| *S.* (*N.*) *aureohirtum* | 49 | 0 | 52 | 0 | 0 | 0 | 0 | 0 | 101 | 3 | 25 |
| *S.* (*N.*) *fruticosum* | 0 | 0 | 9 | 0 | 0 | 0 | 0 | 0 | 9 | 0.3 | 12.5 |
| *S.* (*S.*) *bullatum* | 0 | 0 | 12 | 0 | 0 | 0 | 0 | 0 | 12 | 0.4 | 12.5 |
| *S.* (*S.*) *chamlongi* | 0 | 0 | 0 | 15 | 0 | 0 | 0 | 0 | 15 | 0.4 | 12.5 |
| *S.* (*S.*) *chiangmaiense* | 5 | 0 | 0 | 0 | 0 | 0 | 0 | 0 | 5 | 0.2 | 12.5 |
| *S.* (*S.*) *doipuiense* complex | 2 | 0 | 58 | 0 | 0 | 0 | 0 | 0 | 60 | 1.8 | 25 |
| *S.* (*S.*) *fenestratum* | 48 | 50 | 45 | 64 | 0 | 11 | 0 | 0 | 218 | 6.4 | 62.5 |
| *S.* (*S.*) *grossifilum* | 0 | 0 | 0 | 0 | 0 | 0 | 0 | 4 | 4 | 0.1 | 12.5 |
| *S.* (*S.*) *lampangense* | 0 | 37 | 0 | 0 | 0 | 0 | 0 | 0 | 37 | 1.1 | 12.5 |
| *S.* (*S.*) *nakhonense* | 96 | 0 | 0 | 118 | 62 | 60 | 133 | 79 | 548 | 16.1 | 75 |
| *S.* (*S.*) *nobile* | 0 | 0 | 0 | 0 | 0 | 0 | 38 | 59 | 97 | 2.9 | 25 |
| *S.* (*S.*) *nodosum* | 52 | 8 | 16 | 110 | 0 | 0 | 0 | 0 | 186 | 5.5 | 50 |
| *S.* (*S.*) *prayongi* | 0 | 35 | 0 | 0 | 0 | 0 | 0 | 0 | 35 | 1 | 12.5 |
| *S.* (*S.*) *quinquestriatum* | 47 | 0 | 0 | 0 | 37 | 2 | 0 | 16 | 102 | 3 | 50 |
| *S.* (*S.*) *takense* | 0 | 69 | 0 | 0 | 0 | 0 | 0 | 0 | 69 | 2 | 12.5 |
| *S.* (*S.*) *tani* complex | 7 | 0 | 0 | 10 | 19 | 19 | 30 | 12 | 97 | 2.9 | 75 |
| *S.* (*S.*) *thailandicum* | 69 | 0 | 0 | 0 | 0 | 0 | 0 | 0 | 69 | 2 | 12.5 |
| *S.* (*S.*) *yuphae* | 8 | 0 | 27 | 11 | 0 | 0 | 0 | 0 | 46 | 1.4 | 37.5 |
| *S.* (*S.*) *weji* | 0 | 286 | 120 | 0 | 0 | 0 | 0 | 0 | 406 | 12 | 25 |
| **Total** | **476** | **485** | **396** | **497** | **322** | **332** | **536** | **351** | **3395** | **100.00** |  |
