# Supplementary material for: Biodiversity, seasonal abundance, and distribution of blackflies (Diptera: Simuliidae) in six different regions of Thailand
Source: Parasit Vectors. 2017 Nov 21;10:574. doi: 10.1186/s13071-017-2492-y (PMC5697434; doi:10.1186/s13071-017-2492-y)
Supplement: Supplementary file 9 — Regional distribution and relative abundance of blackflies at 8 sampling sites in southern Thailand. (DOCX 19 kb) [file 13071_2017_2492_MOESM9_ESM.docx]

**Additional file 9: Table S9.** Regional distribution and relative abundance of blackflies at 8 sampling sites in southern Thailand

| **Species** | **Sampling site No.** | | | | | | | | | | |
| --- | --- | --- | --- | --- | --- | --- | --- | --- | --- | --- | --- |
|  | **51** | **52** | **53** | **54** | **55** | **56** | **57** | **58** | **Total** | **%flies** | **%SO** |
| *S.* (*D.*) *pahangense* | 0 | 0 | 0 | 0 | 3 | 0 | 0 | 0 | 3 | 0.1 | 12.5 |
| *S.* (*G.*) *angulistylum* complex | 0 | 23 | 12 | 2 | 0 | 5 | 0 | 4 | 46 | 1.9 | 62.5 |
| *S.* (*G.*) *asakoae* complex | 28 | 25 | 25 | 3 | 33 | 5 | 0 | 5 | 124 | 5.2 | 87.5 |
| *S.* (*G.*) *burtoni* | 65 | 4 | 64 | 91 | 9 | 20 | 205 | 16 | 474 | 19.8 | 100 |
| *S.* (*G.*) *chumpornense* | 6 | 16 | 0 | 0 | 0 | 0 | 6 | 0 | 28 | 1.1 | 37.5 |
| *S.* (*G.*) *sheilae* | 61 | 77 | 67 | 25 | 33 | 13 | 12 | 1 | 289 | 12.1 | 100 |
| *S.* (*G.*) *siamense* complex | 20 | 0 | 8 | 0 | 6 | 0 | 0 | 0 | 34 | 1.4 | 37.5 |
| *S.* (*N.*) *aureohirtum* | 0 | 12 | 0 | 0 | 0 | 0 | 0 | 0 | 12 | 0.5 | 12.5 |
| *S.* (*S.*) *brevipar* | 0 | 0 | 0 | 0 | 16 | 0 | 0 | 0 | 16 | 0.7 | 12.5 |
| *S.* (*S.*) *fenestratum* | 32 | 12 | 18 | 11 | 0 | 5 | 10 | 8 | 96 | 4 | 87.5 |
| *S.* (*S.*) *grossifilum* | 0 | 0 | 0 | 0 | 11 | 0 | 0 | 0 | 11 | 0.5 | 12.5 |
| *S.* (*S.*) *malayense* | 0 | 0 | 0 | 0 | 21 | 0 | 0 | 0 | 21 | 0.9 | 12.5 |
| *S.* (*S.*) *nakhonense* | 18 | 55 | 32 | 41 | 8 | 32 | 85 | 26 | 297 | 12.4 | 100 |
| *S.* (*S.*) *nobile* | 103 | 38 | 48 | 91 | 25 | 211 | 85 | 145 | 746 | 31.1 | 100 |
| *S.* (*S.*) *tani* complex | 0 | 0 | 0 | 14 | 0 | 24 | 15 | 34 | 87 | 3.6 | 50 |
| *S.* (*S.*) *thailandicum* | 0 | 0 | 0 | 8 | 0 | 35 | 4 | 29 | 76 | 3.2 | 50 |
| *S.* (*S.*) *yongi* | 0 | 0 | 0 | 0 | 36 | 0 | 0 | 0 | 36 | 1.5 | 12.5 |
| **Total** | **333** | **262** | **274** | **286** | **201** | **350** | **422** | **268** | **2396** | **100.00** |  |
